# How Attention Changes in Response to Carbohydrate Mouth Rinsing

**DOI:** 10.3390/nu15133053

**Published:** 2023-07-06

**Authors:** Thomas J. Hosang, Sylvain Laborde, Andreas Löw, Michael Sprengel, Niels Baum, Thomas Jacobsen

**Affiliations:** 1 Experimental Psychology Unit, Faculty of Humanities and Social Sciences, Helmut Schmidt University/University of the Federal Armed Forces Hamburg, 22043 Hamburg, Germany; loew@hsu-hh.de (A.L.); sprengem@hsu-hh.de (M.S.); jacobsen@hsu-hh.de (T.J.); 2 Department of Performance Psychology, Institute of Psychology, German Sport University Cologne, 50933 Cologne, Germany; s.laborde@dshs-koeln.de (S.L.);

**Keywords:** carbohydrate mouth rinsing, electroencephalography (EEG), event-related potentials (ERP), reward, sweet taste hedonics, fasting, visuospatial attention, cognition, (intermittent) fasting

## Abstract

Research investigating the effects of carbohydrate (CHO) mouth rinsing on neurocognitive functions is currently limited and has yielded inconsistent results. In this study, we employed the event-related potential (ERP) electroencephalography technique to investigate the effect of CHO mouth rinsing on electrophysiological correlates of visuospatial attention. Using a double-blind, non-nutritive sweetener (NNS)-controlled, within-subjects design, 53 young adults performed a standard cognitive task (modified Simon task) on two separate days in a fasted state (16 h). Intermittently, mouth rinsing was performed either with a CHO (glucose, 18%, 30 mL) or an NNS solution (aspartame, 0.05%, 30 mL). Results revealed that relative to NNS, electrophysiological correlates of both more bottom-up controlled visuospatial attention (N1_pc_-ERP component) were decreased in response to CHO rinsing. In contrast, compared to NNS, more top-down controlled visuospatial attention (N2_pc_-ERP component) was increased after CHO rinsing. Behavioral performance, however, was not affected by mouth rinsing. Our findings suggest that orosensory signals can impact neurocognitive processes of visuospatial attention in a fasted state. This may suggest a central mechanism underlying the ergogenic effects of carbohydrate mouth rinsing on endurance performance could involve modulations of attentional factors. Methodologically, our study underlines that understanding the effects of carbohydrate mouth rinsing at the central level may require combining neuroscientific methods and manipulations of nutritional states.

## 1. Introduction

One intriguing finding from exercise science is the potential of orosensory signals to influence human physical performance [1,2,3,4]. Most of this work has focused on one specific question: Can oral carbohydrate (CHO) availability enhance endurance performance? Current evidence suggests this might be the case for, at least, high-intensity endurance tasks of ≥1 h duration [2,3,4,5,6]. Although oral CHO availability is generally believed to exert its ergogenic effects via central factors [2,3,7,8], one (if not the) fundamental question remains unsolved: What is the exact nature of such a presumed central mechanism?

This study examined the effects of oral CHO availability on neurocognitive processes. This way, we wanted to contribute to the research literature in two respects. First, understanding how oral CHO availability affects human information processing may shed light on neurocognitive factors involved in the putative central mechanism. Through adopting such a mechanistic approach, we may gain a more nuanced understanding of how oral carbohydrate (CHO) availability impacts physical performance at a more general level. Ultimately, this might contribute to the development of evidence-based guidelines for practical applications [5].

Second, by studying the influence of oral CHO availability on human information processing, we may further contribute to the lately intensely investigated question of how reward and cognition interact in humans [9,10] at the methodological level. To the best of our knowledge, we are the first to deliberately apply the most common reward induction protocol in animal affective neuroscience (i.e., administering sweet taste in a state of physiological hunger) [11] to investigate reward/cognition interactions in humans. Therefore, applying our present reward protocol might help better map future animal and human research findings.

At the methodological level, studies examining the effects of oral CHO availability typically apply a technique referred to as *carbohydrate mouth rinsing* [2,3,4]. This method entails the brief rinsing of the oral cavity with a solution containing dissolved CHO, followed by the expulsion of the solution. This approach intends to elicit CHO availability within the oral cavity while concurrently preventing or minimizing post-oral availability. Based on the assumption that this technique mainly induces oral availability, it is currently widely posited that the ergogenic effects of CHO mouth rinsing on endurance performance are due to central rather than peripheral factors.

Assuming that CHO mouth rinsing exerts its ergogenic effects via a central mechanism, it is reasonable to hypothesize that it may also enhance cognitive functions. Critically, neurocognitive factors involved in a potential central mechanism might be identified by investigating the effects of CHO mouth rinsing on information processing. The question remains, however, as to how these alterations in neurocognitive processes can be effectively measured and analyzed. 

One straightforward approach to examining the impact of CHO mouth rinsing on cognitive performance is to assess behavioral outcomes, such as response times (RTs) and accuracy rates while performing a cognitive task. However, this methodology has limitations in its ability to fully capture mental processes that may not be readily observable at the behavioral level. 

Analyzing the summed electrical brain activity using electroencephalography (EEG) is another way to infer mental processes more granularly. By applying the event-related potential (ERP) technique to the EEG data, which involves averaging the data time-locked to a specific event, it is possible to assess neural responses associated with specific mental processes [12]. The EEG-ERP technique possesses two key strengths. First, it can capture rapid cognitive processes in the time domain with high temporal resolution, down to milliseconds [12]. Second, it enables a relatively precise differentiation between subprocesses involved in the perception–action cycle, which cannot be achieved to the same extent through behavioral measures alone [12]. As such, by applying this technique, the potential effects of CHO mouth rinsing on otherwise non-observable mental processes can ultimately be captured with high precision in the time domain. 

Despite the potential advantages of using EEG-ERP to study the effects of CHO mouth rinsing on neurocognitive processes, to our knowledge, only three studies have investigated this topic [13,14,15]. Among these, a seminal study investigated whether CHO mouth rinsing might benefit human cognition [14]. In this study, participants performed two consecutive Stroop tasks [16] on three separate occasions. In addition, a single mouth rinse was performed between the Stroop tasks during each session (CHO, caffeine, or neutral solution). Behavioral task performance was measured in terms of RTs and accuracy rates. In addition to behavioral task performance, the P3-ERP component was assessed, commonly viewed as an indicator of context updating [17]. However, neither behavioral performance nor the P3-ERP component showed any significant effects of CHO mouth rinsing.

More recently, another study [13] used the EEG-ERP method to investigate whether CHO mouth rinsing enhances mental processes. Participants performed a cognitive testing battery twice on two separate days. A single mouth rinse (CHO or non-nutritive sweetener [NNS] solution) was conducted between batteries. In addition to the P3-ERP component, an electrophysiological correlate of error processing termed error-related negativity (N_e_/ERN [18,19]) was further assessed. While the N_e_/ERN-ERP component was not affected by CHO mouth rinsing, P3 latency, elicited in a Flanker task [20], decreased from pre- to post-rinsing in response to NNS rinsing, but not CHO rinsing. Moreover, RTs during the Flanker task increased after CHO rinsing but not NNS rinsing. 

In our recent study [15], we used the EEG-ERP method to investigate whether CHO mouth rinsing affects mental processing through reward circuits [7,8,21]. We assessed the N_e_/ERN-ERP component, which has been shown to be susceptible to reward manipulation [9,10,22]. To sensitize brain reward circuits and elicit a strong reward response [11,23], we induced physiological appetite through a preceding 16 h fasting period. Participants rinsed their mouths with a CHO or an NNS solution while performing a modified Flanker task. Our results revealed decreased N_e_/ERN-ERP component amplitudes in response to CHO rinsing relative to NNS rinsing. We interpreted our findings within an influential reward framework that distinguishes between psychological reward components of ‘liking’ (core affective reaction) and ‘wanting’ (incentive motivation to acquire reward) [11,24] and concluded that CHO mouth rinsing might have impaired error processing via the 'liking' component of reward [15].

Given this background, it is challenging to draw clear overall conclusions regarding the impact of CHO mouth rinsing on electrophysiological correlates of cognition. However, a commonality across all studies is the lack of evidence supporting CHO mouth rinsing enhancing mental processes. Instead, the opposite may hold true; CHO mouth rinsing potentially has a negative impact on electrophysiological correlates of cognitive processes related to error processing [15] and attention [13]. 

In the current EEG-ERP study, we aimed to investigate the potential effects of CHO mouth rinsing on human cognition, specifically focusing on its impact on electrophysiological correlates of visuospatial attention. To achieve this goal, we applied a standard cognitive task known as a Simon task [25]. The Simon task involves lateralized (visual) target presentations, with the non-spatial stimulus dimension (e.g., color or shape) indicating the required rightward or leftward response. Despite being irrelevant to task performance, the target’s spatial location (left or right hemifield) often influences performance in a particular way, known as the Simon effect. Congruent trials, where the spatial location of the required response and the spatial location of the stimulus overlap, generally result in improved behavioral performance. Conversely, non-overlapping conditions, known as incongruent trials, typically lead to a decrease in behavioral performance. 

Using such a task, subprocesses of visuospatial attention can be assessed using the EEG-ERP method. More automatic bottom-up controlled visuospatial attentional processes can be investigated by analyzing the N1-posterior-contralateral (N1_pc_ [26,27,28])-ERP component. On the other hand, more top-down controlled visuospatial attentional processes can be examined through an ERP component termed N2-posterior-contralateral (N2_pc_ [29,30]). Taken together, we investigated the effects of CHO mouth rinsing on behavioral performance (RTs and proportions of correct responses [%correct]) and electrophysiological correlates of visuospatial attention (N1_pc_ and N2_pc_-ERP component amplitudes). Based on the notion that neural reward circuits might mediate the central effects of CHO mouth rinsing [7,8,21], physiological appetite was induced by fasting to sensitize neural reward circuits [11,23]. Our study was exploratory, yet based on previous results [15]. We hypothesized that CHO mouth rinsing, relative to NNS rinsing, would be more likely to attenuate visuospatial attention, possibly involving the 'liking' component of reward.

The aim of the present study was two-fold: First, to shed light on an often-suspected central mechanism thought to underlie the ergogenic effects of CHO mouth rinsing on physical performance by understanding how cognition might be affected by CHO mouth rinsing. Second, understanding how CHO mouth rinsing affects human cognition might contribute to the research field on the interaction between (orosensory) reward and cognition in humans.

## 2. Materials and Methods

### 2.1. Participants

Some of the data collected within the context of this study have been published elsewhere [15]. While the present manuscript is specifically devoted to data derived from a modified Simon task, the previous publication exclusively concerns data collected during a modified Flanker task. Therefore, the sample evaluated in the present manuscript (*N* = 61) is consistent with that of [15], with a few notable exceptions. Three participants were excluded due to technical difficulties with the Simon task presentation/recording, three did not comply with button press instructions (i.e., confounded button press assignments), and two had accuracy rates below 50% on at least one condition. The data of 53 subjects were eligible for further processing (sex: [f = 19, m = 34], handedness: [left = 5, ambidextrous = 2, right = 46], age (years): [*M* = 26.17, *SD* = 3.88], height (cm): [*M* = 178.43, *SD* = 7.3], BMI (kg/m^2^): [*M* = 23.22, *SD* = 2.07]). All participants reported being non-diabetic, had normal or corrected-to-normal vision, and gave written informed consent before study participation. The present study agreed with the ethical code of the World Medical Association and received approval from the German Sport University’s ethics committee (091/2016). Participants were unaware of the aim of the study before engagement.

### 2.2. Study Design

A mixed-factorial design was applied. Factors were UTILITY (levels: high vs. low; between factor), SOLUTION (levels: CHO vs. NNS; within factor), and CONGRUENCY (levels: congruent vs. incongruent; within factor). Please note that we originally intended to apply a within-factorial design with the factors SOLUTION (levels: CHO vs. NNS; within factor) and CONGRUENCY (levels: congruent vs. incongruent; within factor). However, due to two reasons, the actual design of this study deviated from the planned one. (1) As previously described and discussed in [15], placebo control failed, as evidenced by insufficient taste-matching of the solutions (i.e., solutions were perceived differently at the subjective level). For this reason, we refrain from using the term “placebo-controlled” in the further course and instead use the word “NNS-controlled”. (2) The counterbalancing of response mappings was counteracted by an error that occurred during the programming of the Simon task, which was only identified during the analysis of the present dataset. We initially planned fixed proportions of the factor CONGRUENCY (congruent: 75% vs. incongruent: 25%) across participants. However, due to the mentioned programming error, different response mappings were associated with varying proportions of congruency ((a) congruent: 75% vs. incongruent: 25%; (b) congruent: 25% vs. incongruent: 75%). Thus, the programming error (a) prevented counterbalancing of the response mapping and (b) introduced an a priori unintended experimental between-subject factor of UTILITY [31,32,33]. Manipulations of congruency proportions are typically applied to investigate adaptive cognitive control [31,32,33,34]. Very simplified, such manipulations can be applied in three ways: (1) Administering blocks of trials with mostly congruent, relative to incongruent trials, often referred to as “high-utility” conditions. (2) Administering blocks of trials with mostly incongruent, relative to congruent trials, often called “low-utility” conditions. (3) Finally, one can also implement blocks of trials with equal proportions of congruent and incongruent trials. As performance is significantly better in congruent trials than incongruent trials, the distractor information can be described as more useful in congruent trials. Therefore, overall distractor utility can be described as higher in the high-utility relative to low-utility conditions. Importantly, high-utility conditions typically result in larger congruency effects relative to low-utility conditions [34], often referred to as “(list-wide) proportion-congruency effect” [32,33,34].

Statistical effects of the factor UTILITY will only be brought up in the discussion in case of interacting with the factor SOLUTION. Otherwise, statistical findings involving the UTILITY factor will only be reported in the results section without further discussion later on. Due to the abovementioned issues, the actual design must be described as a randomized, double-blind, NNS-controlled, mixed design. All experimental conditions and the sum of participants assigned to each latter are reported in the Supplementary Materials.

### 2.3. Apparatus and Stimuli

Python’s PsychoPy package (v1.82) was used for Simon task programming and control [35]. The task was launched from a standard PC, and a 24-inch LED monitor (1920 × 1080-pixel resolution, 144 Hz, 24GM77-B, LG Corp., Seoul, Republic of Korea) was used to present the stimuli visually. A millisecond-resolving button box (4-button response pad, The Black Box ToolKit Ltd., Sheffield, UK) was used for behavioral data recording. Running BrainVision Recorder software (v1.22.0001, Brain Products, Gilching, Germany) on a second PC, EEG data, triggers (coding timing and identity of all task stimuli), and behavioral responses were recorded. The experiment was run in a noise-shielded and artificially lit room. The composition of the taste stimuli was based on previous research [36]. For NNS rinsing, aspartame was used as a tastant (0.05%, UD Chemie, Wörrstadt, Germany). For CHO rinsing, glucose was used as a tastant (18%, MyProtein, Northwich, UK). Tap water was used as a carrier fluid for all solutions. Third parties prepared all solutions without further involvement in the experimental procedure. Solutions were poured into paper cups of 30 mL each, and cups were covered with plastic lids to mask potential visual cues. Subjective ratings of stress, task difficulty, hunger, solutions’ sweetness, solutions’ viscosity, and liking of the solution were assessed via visual analog scales (100 mm; vertical line [37]). In addition, affective valence and arousal were assessed employing the Self-Assessment Manikin scale [38].

### 2.4. Procedure

Participants attended two sessions (separate days; ≥72 h apart), each one of them in a fasted state (16 h). Thereby, we aimed to prevent potential carry-over effects between solutions. Therefore, a Flanker task [15] and a Simon task had to be performed in both sessions. Each session started with the completion of questionnaires (questionnaire time point 1 [TP1]: demographic data, ratings: [hunger, stress, affective valence, arousal]). Subsequently, electroencephalographic (EEG) and electrooculographic (EOG) sensors were mounted on the participants’ heads. Following a short mouth rinsing familiarization, questionnaires had to be filled a second time (questionnaire time point 2 [TP2]: ratings: [hunger, stress, affective valence, arousal, sweetness, viscosity, liking]). 

Then, a Simon or Flanker task was started (the task sequence was kept constant between sessions for each subject). Results concerning the Flanker data have been reported elsewhere [15]. The analysis of the data collected during the Simon task is relevant to the present manuscript. We used a modified version of a standard visual Simon task [25] of 600 trials. The Simon task is reported in full detail in the Supplementary Materials. In brief, each trial involved the visual presentation of a fixation stimulus (shape: circle; RGB color code: [0.5, 0.5, 0.5]; frames: randomly varied from 50 to 80; degrees of visual angle: 0.05°) that was subsequently joined by a target stimulus by a target stimulus (shape: rectangle [vertical or horizontal orientation]; RGB color code: [0.5, 0.5, 0.5]; frames: 10; degrees of visual angle: 2°). Eye-to-monitor distance was ~120 cm. All stimuli were presented on a black screen. Participants were asked to respond as quickly and accurately as possible to the target’s shape orientation. Therefore, they had to press one of two buttons with their left or right index finger. Target stimulus position and orientation could therefore be congruent or incongruent in terms of the spatial location of the target stimulus and the spatial location of the required button press. The Simon task involved an adaptive response deadline to ensure comparable error rates between sessions and participants. The Simon task was preceded and regularly interrupted (every 150 trials) by brief mouth rinsing periods. Each mouth rinse lasted for a duration of 10 s. CHO or NNS was administered as a taste stimulant in one session. Finally, participants were asked to complete a set of questionnaires once more (questionnaire time point 3 [TP3]: ratings: [hunger, stress, affective valence, arousal, sweetness, viscosity, liking, task difficulty]).

### 2.5. EEG Recording

A full report of the EEG recording and processing is available in the Supplementary Materials.

Sixty Ag/AgCl active electrodes were mounted into an elastic cap (actiCAP, Brain Products GmbH, Gilching, Germany) and placed in accord with the 10-10 system at scalp sites FP1, FP2, AF7, AF3, AFz, AF4, AF8, F7, F5, F3, F1, Fz, F2, F4, F6, F8, FT7, FC5, FC3, FC1, FCz, FC2, FC4, FC6, FT8, C5, C3, C1, Cz, C2, C4, C6, TP9, TP7, CP5, CP3, CP1, CPz, CP2, CP4, CP6, TP8, TP10, P7, P5, P3, P1, Pz, P2, P4, P6, P8, PO7, PO3, POz, PO4, PO8, O1, Oz, and O2. Four additional electrodes were used to record horizontal (HEOG) and vertical electrooculograms (VEOG). The VEOG was recorded by placing electrodes above and below the right eye. HEOG was recorded by placing electrodes at the right and left outer canthi. The ground electrode was placed at scalp location Fpz, and electrode Cz was used as the recording reference. The EEG and EOG signal was DC recorded continuously (filter: {type: IIR Butterworth 2nd order causal lowpass; high cutoff frequency: 280 Hz (−3 dB); sampling rate: 1000 Hz; resolution: 0.0488281 µV}). An actiCHamp amplifier (Brain Products, Gilching, Germany) and BrainVision Recorder software (v1.22.0001, Brain Products, Gilching, Germany) were used for recording. Impedances were kept <10 kΩ.

### 2.6. Offline EEG Processing

All offline EEG signal processing (offline processing sampling rate: 1000 Hz) was performed using custom Python scripts, principally utilizing Python’s MNE package [39]. Bad channel detection was performed automatically using PyPREP’s “NoisyChannel” methods [40]. A mixed ANOVA with the factors SOLUTION (levels: CHO vs. NNS; within factor) and UTILITY (levels: high vs. low; between factor) was performed for the “sum of interpolated channels”. Neither a main effect of SOLUTION or UTILTY nor an interaction of SOLUTION × UTILITY was obtained (*F*s < 1). The corresponding ANOVA table and descriptive statistics are reported in the Supplementary Materials. An automated muscle artifact detection was performed using MNE’s “annotate_muscle_zscore” function. Ocular artifact correction was performed via MNE’s independent component analysis [41]. Therefore, ICA decomposition was run on a highpass-filtered (type: IIR Butterworth 4th order zero-phase highpass; lower frequency cutoff: 1 Hz (−6 dB)) and evenly time-segmented (1 s) copy of the raw EEG data. Segments with large voltage deviations (peak-to-peak deviations >500 µV in any EEG channel), non-task periods (pre-/post task & rinsing), as well as bad channels, were ignored during decomposition. Subsequently, MNE’s “find_bads_eog” function automatically detected independent components reflecting vertical or horizontal eye movements. This selection was then visually and independently cross-checked by two experienced EEG researchers. Visual inspection was performed by comparing the timing and shape of the ICA components with the EOG signals and examining the components’ topographies. In the case of no consensus, a third experienced EEG researcher was brought in to contribute to a final decision concerning component selection. A mixed ANOVA with the factors SOLUTION (levels: CHO vs. NNS; within factor) and UTILITY (levels: high vs. low; between factor) was performed for “selected ocular ICA components”. The corresponding ANOVA table and descriptive statistics are reported in the Supplementary Materials. The final component selection was then zeroed out from a notch (type: FIR one-pass, zero-phase, non-causal bandstop; frequencies: [50, 100, 150, 200, 250], filter length: 6601 samples) and bandpass-filtered (type: IIR Butterworth 4th order zero-phase bandpass; lower frequency cutoff: 0.10 Hz (−6 dB); upper cutoff frequency: 30 Hz (−6 dB)) copy of the raw EEG data. Bad channel interpolation was performed using MNE’s spherical spline method. The EEG data were then re-referenced to the average reference. Subsequently, epoching of the continuous data was performed relative to target stimulus onset (epoch interval: −200:600 ms; baseline correction interval: −200:0). Epochs meeting at least one of the following rejection criteria were excluded during epoching: (1) epochs with large voltage deviations (peak-to-peak deviations > 100 µV in any EEG channel), (2) epochs containing previously detected muscle artifacts, (3) epochs representing (post-)error or (post-)miss trials, (4) epochs representing correct trials with outlier RTs (<100 ms or >1000 ms), (5) epochs representing one of the first three trials of each task block, and (6) epochs that exceeded 1.5*interquartile range of subject’s condition mean alpha power averaged across electrodes O1/2, P1/2, P3/4, P5/6, P7/8, PO3/4, and PO7/8. A mixed ANOVA with the factors SOLUTION (levels: CHO vs. NNS; within factor), CONGRUENCY (levels: congruent vs. incongruent; within factor), and UTILITY (levels: high vs. low; between factor) was performed for “sum of trials included into statistical analysis”. Relevant to the current analysis, neither a main effect of SOLUTION nor interactions of SOLUTION × UTILITY, SOLUTION × CONGRUENCY, or CONGRUENCY × SOLUTION × UTILITY (*F*s < 1) were obtained. See the Supplementary Materials for a full report of the corresponding statistical analysis. Further, a table is included in the Supplementary Materials reporting the single-subject trial counts. The continuous epochs were then averaged for all conditions. Lateralized ERPs were calculated as difference waves. Therefore, ipsilateral electrode activity was subtracted from that of contralateral electrode activity, both relative to the target stimulus position, and then averaged across left and right hemispheres; i.e., [(left hemisphere electrodes−right hemisphere electrodes)_right-hemifield target stimulus_ + (right hemisphere electrodes−left hemisphere electrodes)_left-hemifield target stimulus_]/2. These difference waves were then averaged across all posterior and occipital electrodes (O1/2, P1/2, P3/4, P5/6, P7/8, PO3/4, PO7/8 [41]). N1_pc_ and N2_pc_-ERP components were quantified as mean amplitudes. We adopted a variant of the collapsed localizer technique [42] to identify appropriate time windows for averaging. Therefore, we computed a collapsed grand average waveform by averaging the ERPs across all conditions. The average component-time course was defined as the time window ranging from the time point of the left to the time point of the right local minimum surrounding the component’s peak (see Supplementary Materials for the corresponding collapsed localizer waveform plot). These time windows were ultimately used to quantify the N1_pc_ and N2_pc_-ERP components for each participant. 

For the statistical analysis of the N1_pc_ and N2_pc_-ERP component, mixed ANOVAs with UTILITY (levels: high vs. low; between factor), SOLUTION (levels: CHO vs. NNS; within factor), and CONGRUENCY (levels: congruent vs. incongruent; within factor) were performed.

### 2.7. Behavioral Data Analysis

Behavioral data analysis focused on RTs and %correct. The same trials used for the ERP analysis were used for the analysis of RTs. For the analysis of %correct, percentages were calculated in relation to those incorrect trials that were not rejected during EEG preprocessing. For the analysis of RTs and %correct, mixed ANOVAs with the factors UTILITY (levels: high vs. low; between factor), SOLUTION (levels: CHO vs. NNS; within factor), and CONGRUENCY (levels: congruent vs. incongruent; within factor) were performed.

### 2.8. Questionnaire and Solution Weight Analysis

Mixed ANOVAs with the factors UTILITY (levels: high vs. low; between factor), SOLUTION (levels: CHO vs. NNS; within factor), and TIME POINT (TP) (levels: TP1 vs. TP2 vs. TP3; within factor) were conducted for the analysis of the subjective ratings of stress, hunger, affective valence, and arousal. Mixed ANOVAs with the factors UTILITY (levels: high vs. low; between factor), SOLUTION (levels: CHO vs. NNS; within factor), and TP (levels: TP2 vs. TP3; within factor) were performed for the analysis of the subjective ratings of sweetness, viscosity, and liking. In addition, a mixed ANOVA with the factors UTILITY (levels: high vs. low; between factor) and SOLUTION (levels: CHO vs. NNS; within factor) was conducted to analyze perceived task difficulty. Finally, a mixed ANOVA with the factors UTILITY (levels: high vs. low; between factor), SOLUTION (levels: CHO vs. NNS; within factor), and TP (levels: pre vs. post; within factor) was performed for the weight of the solution.

### 2.9. Statistics

The Greenhouse–Geisser correction was performed in case of violation of sphericity. Significance levels were set at *p* < 0.05. Post hoc *t*-test comparisons were controlled via the Holm–Bonferroni method. All post hoc *t*-test *p* values are reported as Holm–Bonferroni-corrected values. All statistical tests reported below are available in tabular form in the Supplementary Materials. All descriptive statistics are reported in the Supplementary Materials. Please note that post hoc *t*-tests are only reported in cases of statistical significance within the manuscript. All non-significant post hoc *t*-tests are reported in the Supplementary Materials. All statistical analyses were performed using GNU R’s afex and emmeans packages. Further, all analyses were cross-checked using JASP. To maintain conciseness and readability, only statistically significant results will be reported in the body of the manuscript. However, for comprehensive transparency and thoroughness, all statistical analyses, including those that did not yield statistically significant results, are presented in full detail in the Supplementary Materials of the manuscript.

## 3. Results

### 3.1. Manipulation Checks

*Fasting*: All participants self-reported that they had fasted for 16 h before all testing sessions. 

*Weight of solution:* A main effect of SOLUTION was obtained (*F*(1, 51) = 56.83, *p* < 0.001, η_p_^2^ = 0.53), revealing lower weight for the NNS solution (*M* = 36.91, *SD* = 1.98) compared to the CHO solution (*M* = 38.69, *SD* = 1.82). The difference in weight between the two solutions is most likely a result of their disparate densities. 

### 3.2. Questionnaire Ratings

#### 3.2.1. Hunger

A main effect of TP was observed (*F*(2,102) = 9.77, *p* < 0.001, η_p_^2^ = 0.16). Post hoc *t*-tests revealed an increase in hunger from TP1 (*M* = 5.45, *SD* = 2.81) to TP3 (*M* = 6.09, *SD* = 3.1) (*t*(51) = −2.41, *p* = 0.04, *d* = −0.21) as well as an increase from TP2 (*M* = 5.06, *SD* = 2.95) to TP3 (*t*(51) = −4.66, *p* < 0.001, *d* = −0.35), which was likely due to our fasting manipulation. Further, an interaction of SOLUTION × TP (*F*(1.59, 80.95) = 4.6, *p* = 0.019, η_p_^2^ = 0.08) was observed. Here, for the NNS condition, hunger increased from TP1 (*M* = 5.15, *SD* = 2.92) to TP3 (*M* = 6.4, *SD* = 2.92) (*t*(51) = −4.3, *p* = 0.001, *d* = −0.42), as well as from TP2 (*M* = 4.87, *SD* = 2.86) to TP3 (*t*(51) = −4.62, *p* < 0.001, *d* = −0.51). 

#### 3.2.2. Stress

Statistical analysis revealed a main effect of TP (*F*(1.51, 76.86) = 14.17, *p* < 0.001, η_p_^2^ = 0.22). Here, post hoc *t*-tests revealed an overall increase in stress from TP1 (*M* = 1.84, *SD* = 1.63) to TP3 (*M* = 2.84, *SD* = 2.51) (*t*(51) = −3.77, *p* = 0.001, *d* = −0.53), as well as from TP2 (*M* = 1.74, *SD* = 1.59) to TP3 (*t*(51) = −4.36, *p* < 0.001, *d* = -.58). Further, a main effect of UTILITY (*F*(1, 51) = 4.91, *p* = 0.031, η_p_^2^ = 0.09) was obtained, revealing higher stress values for the low-utility group (*M* = 2.56, *SD* = 2.18) compared to the high-utility group (*M* = 1.73, *SD* = 1.75). Additionally, an interaction of TP × UTILITY (*F*(1.51, 76.86) = 4.57, *p* = 0.022, η_p_^2^ = 0.08). Post hoc *t*-tests revealed that for the low-utility group, there was an increase in stress from TP1 (*M* = 2.12, *SD* = 1.66) to TP3 (*M* = 3.67, *SD* = 2.63) (*t*(51) = −4.06, *p* = 0.001, *d* = −0.81), as well as from TP2 (*M* = 1.91, *SD* = 1.71) to TP3 (*t*(51) = −4.85, *p* < 0.001, *d* = −0.92). Further, comparing both groups, stress values at TP3 were higher for the low-utility group compared to the high-utility group (*M* = 2.04, *SD* = 2.13) (*t*(51) = 2.83, *p* = 0.046, *d* = 0.85).

#### 3.2.3. Arousal

Statistical analysis revealed a main effect of UTILITY (*F*(1, 51) = 6.08, *p* = 0.017, η_p_^2^ = 0.11). Higher arousal values were obtained for the low-utility group (*M* = 3.46, *SD* = 1.73) compared to the high-utility group (*M* = 2.72, *SD* = 1.73). 

#### 3.2.4. Affective Valence

We obtained a main effect of TP (*F*(1.77, 90.13) = 13.02, *p* < 0.001, η_p_^2^ = 0.2). Post hoc *t*-tests revealed a decrease in affective valence from TP1 (*M* = 6.68, *SD* = 1.6) to TP3 (*M* = 6, *SD* = 1.81) (*t*(51) = 4.28, *p* < 0.001, *d* = 0.42), as well as from TP2 (*M* = 6.64, *SD* = 1.58) to TP3 (*t*(51) = 3.87, *p* = 0.001, *d* = 0.39). Additionally, an interaction of TP × UTILITY was obtained (*F*(1.77, 90.13) = 4.35, *p* = 0.02, η_p_^2^ = 0.08). Post hoc *t*-tests revealed that for the low-utility group, there was a decrease in affective valence from TP1 (*M* = 6.67, *SD* = 1.69) to TP3 (*M* = 5.54, *SD* = 1.81) (*t*(51) = 4.92, *p* < 0.001, *d* = 0.68), as well as from TP2 (*M* = 6.46, *SD* = 1.65) to TP3 (*t*(51) = 3.84, *p* = 0.003, *d* = 0.56).

#### 3.2.5. Sweetness

A main effect of SOLUTION was observed (*F*(1, 51) = 48.54, *p* < 0.001, η_p_^2^ = 0.49), revealing higher sweetness scores for the CHO condition (*M* = 7.81, *SD* = 1.43) compared to NNS (*M* = 5.32, *SD* = 2.56). 

#### 3.2.6. Viscosity

Statistical analysis revealed a main effect of SOLUTION (*F*(1, 51) = 9.44, *p* = 0.003, η_p_^2^ = 0.16), revealing that the CHO condition was perceived as more viscous (*M* = 7.71, *SD* = 1.99) compared to the NNS condition (*M* = 8.35, *SD* = 1.59). 

#### 3.2.7. Liking

Statistical analysis did not reveal any significant results.

#### 3.2.8. Task Difficulty

Statistical analysis did not reveal any significant results.

### 3.3. Response Times (RTs)

**Figure 1:**
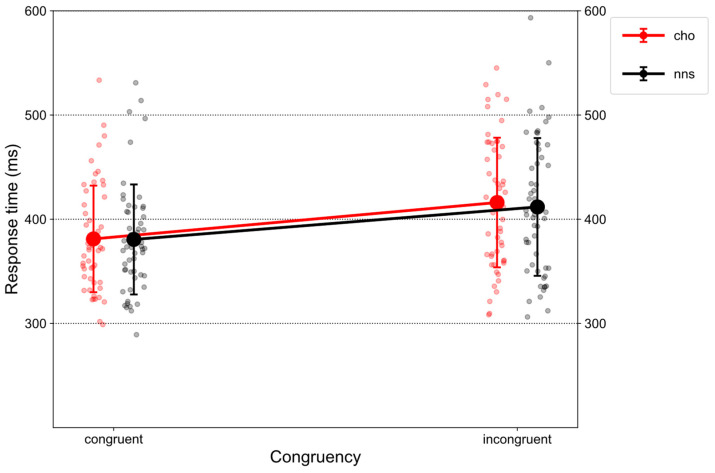
Mean response times (RTs) displayed for congruent and incongruent trials in response to CHO and NNS mouth rinsing. The participants’ mean values are shown as scattered circles surrounding the respective condition’s mean value. Error bars represent one standard deviation. Abbreviations: carbohydrate (CHO), non-nutritive sweetener (NNS).

Figure 1 illustrates RTs on congruent and incongruent trials in response to CHO and NNS rinsing.

There was a main effect of CONGRUENCY (*F*(1, 51) = 193.43, *p* < 0.001, η_p_^2^ = 0.79), revealing an overall congruency effect, i.e., larger RTs on incongruent trials (*M* = 413.91, *SD* = 63.93) compared to congruent ones (*M* = 380.85, *SD* = 51.74). Further, a main effect of UTILITY was obtained (*F*(1, 51) = 4.89, *p* = 0.031, η_p_^2^ = 0.09). Overall, larger RTs were observed for the high-utility group (*M* = 412.49, *SD* = 59.64) compared to the low-utility group (*M* = 382.83, *SD* = 57.61). Additionally, an interaction of CONGRUENCY × UTILITY (*F*(1, 51) = 232.35, *p* < 0.001, η_p_^2^ = 0.82) was observed. Post hoc *t*-tests revealed a congruency effect for the high-utility group (*t*(51) = 20.421, *p* < 0.001, *d* = 1.32), i.e., larger RTs on incongruent trials (*M* = 447.88, *SD* = 53.64) compared to congruent trials (*M* = 377.11, *SD* = 41.87). Further, on incongruent trials (*t*(51) = 4.81, *p* < 0.001, *d* = 1.24), larger RTs were observed for the high-utility group (*M* = 447.88, *SD* = 53.64) compared to the low-utility group (*M* = 381.21, SD = 55.73). 

### 3.4. Proportions of Correct Responses (%Correct)

**Figure 2:**
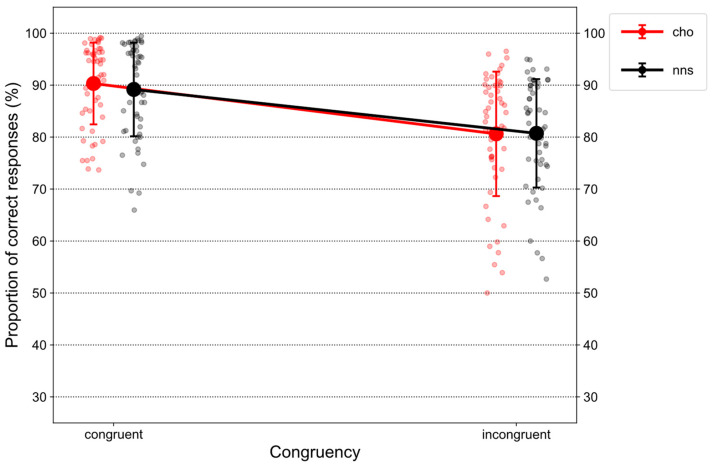
Mean proportions of correct responses (%correct) displayed for congruent and incongruent trials in response to CHO and NNS mouth rinsing. The participants’ mean values are shown as scattered circles surrounding the respective condition’s mean value. Error bars represent one standard deviation. Abbreviations: carbohydrate (CHO), non-nutritive sweetener (NNS).

Figure 2 illustrates %correct on congruent and incongruent trials in response to CHO and NNS rinsing.

Statistical analysis revealed a main effect of CONGRUENCY (*F*(1, 51) = 79.32, *p* < 0.001, η_p_^2^ = 0.61) that was characterized by overall lower proportions of correct responses on incongruent trials (*M* = 80.68, *SD* = 11.18) compared to congruent trials (*M* = 89.74, *SD* = 8.44). Additionally, an interaction of CONGRUENCY × UTILITY (*F*(1, 51) = 215.3, *p* < 0.001, η_p_^2^ = 0.81) was observed. Post hoc *t*-tests revealed a congruency effect for the high-utility group (*t*(51) = −16.52, *p* < 0.001, *d* = 4.03), i.e., lower proportions of correct responses on incongruent trials (*M* = 71.91, *SD* = 9.31) compared to congruent trials (*M* = 96.69, *SD* = 1.86). In contrast, a “reversed” congruency effect was observed for the low-utility group (*t*(51) = 4.12, *p* < 0.001, *d* = 0.99), i.e., lower proportions of correct responses on congruent trials (*M* = 83.06, *SD* = 6.70) compared to incongruent trials (*M* = 89.12, *SD* = 4.03). Further, comparing both groups on congruent trials (*t*(51) = 13.13, *p* < 0.001, *d* = 2.22), lower proportions of correct responses were observed for the low-utility group compared to the high-utility group. Finally, comparing both groups on incongruent trials, the opposite pattern was obtained (*t*(51) = 9.94, *p* < 0.001, *d* = 2.8). Here, lower proportions of correct responses were observed for the high-utility group compared to the low-utility group. 

### 3.5. N1_pc_-ERP Component

**Figure 3:**
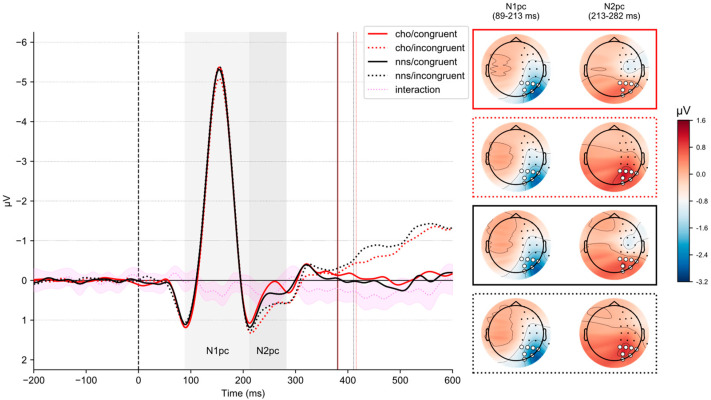
(**Left**): Grand average of target-locked ERP waveforms for all SOLUTION × CONGRUENCY factor combinations. The dotted, violet-colored line further depicts the difference wave for the SOLUTION × CONGRUENCY interaction ((CHO_incongruent_ − CHO_congruent_) − (NNS_incongruent_ − NNS_congruent_)). The surrounding violet area depicts the latter’s difference wave 0.95 confidence interval. LERP waveforms are averaged across electrodes O1/2, P1/2, P3/4, P5/6, P7/8, PO3/4, and PO7/8. Post-target onset vertical lines depict the mean RTs corresponding to the waveforms. (**Right**): Topographical maps averaged for the time windows used for component scoring. The displayed data are re-referenced to the average reference and baseline corrected between −200 and 0 ms. Abbreviations: N1 posterior contralateral (N1_pc_), N2 posterior contralateral (N2_pc_), carbohydrate (CHO), non-nutritive sweetener (NNS).

Figure 3 illustrates lateralized ERPs on congruent and incongruent trials in response to CHO and NNS rinsing. The corresponding grand average waveforms for all sensors are reported in the Supplementary Materials.

Statistical analysis revealed an interaction of CONGRUENCY × UTILITY (*F*(1, 51) = 4.25, *p* = 0.044, η_p_^2^ = 0.08). Post hoc *t*-tests revealed no significant differences (all *p*s > 0.3). Statistical analysis revealed an interaction of SOLUTION × CONGRUENCY (*F*(1, 51) = 9.04, *p* = 0.004, η_p_^2^ = 0.15). Here, post hoc *t*-tests revealed smaller N1_pc_-ERP component amplitudes on incongruent trials comparing the CHO condition (*M* = −1.7, *SD* = 1.03) to the NNS condition (*M* = −1.88, *SD* = 1.24) (*t*(51) = 2.88, *p* = 0.023, *d* = 0.15).

### 3.6. N2_pc_-ERP Component

Statistical analysis revealed a main effect of CONGRUENCY (*F*(1, 51) = 15.05, *p* < 0.001, η_p_^2^ = 0.23), revealing larger N2_pc_-ERP component amplitudes on congruent trials (*M* = 0.44, *SD* = 1.08) compared to incongruent ones (*M* = 0.80, *SD* = 1.02). Further, an interaction of CONGRUENCY × UTILITY (*F*(1, 51) = 20.71, *p* < 0.001, η_p_^2^ = 0.29) was obtained. Post hoc *t*-tests revealed that for the low-utility group (*t*(51) = 6.02, *p* < 0.001, *d* = 0.75), N2_pc_-ERP component amplitudes were larger on congruent trials (*M* = 0.05, *SD* = 1.03) compared to incongruent trials (*M* = 0.82, *SD* = 1.12). Further, in congruent trials (*t*(51) = −2.98, *p* = 0.0131, *d* = −0.78), larger N2_pc_-ERP component amplitudes were obtained for the low-utility group (*M* = 0.05, *SD* = 1.03) compared to the high-utility group (*M* = 0.85, *SD* = 0.98). Additionally, an interaction of SOLUTION × CONGRUENCY (*F*(1, 51) = 5.88, *p* = 0.019, η_p_^2^ = 0.1) was observed (see Figure 3). Post hoc *t*-tests revealed that for the CHO condition (*t*(51) = 4.79, *p* < 0.001, *d* = 0.48), larger N2_pc_-ERP component amplitudes were observed on congruent trials (*M* = 0.36, *SD* = 1.12) compared to incongruent trials (*M* = 0.85, *SD* = 0.97).

## 4. Discussion

Previous research on the potential effects of CHO mouth rinsing on electrophysiological correlates of neurocognitive functions is scarce and largely inconclusive [13,14,15]. In this study, we investigated whether CHO mouth rinsing might affect electrophysiological correlates of visuospatial attention in a state of physiological appetite [11,23] (induced by 16 h of fasting). Our findings show that depending on whether a CHO or an NNS solution is orally rinsed, electrophysiological correlates of more bottom-up and top-down controlled visuospatial attention can be affected differently. In contrast, the type of solution rinsed did not affect behavioral measures such as RTs and %correct. Our results emphasize the importance of utilizing neuroscientific methods and manipulating physiological appetite to fully understand the effects of CHO mouth rinsing at the central level. Furthermore, our findings suggest that previously observed ergogenic effects of CHO mouth rinsing on endurance performance [2,3,4] may involve alterations in attentional processes.

The main findings of the present study concern our electrophysiological brain measures. Here, N1_pc_ and N2_pc_-ERP component amplitudes were affected by whether mouth rinsing was performed with a CHO or the NNS solution as a function of congruency.

On the one hand, smaller N1_pc_-ERP component amplitudes were observed in response to CHO mouth rinsing relative to NNS rinsing. Importantly, this effect was only observed for incongruent trials. Previous research has suggested that N1_pc_-ERP component amplitude may reflect the initial orienting of attention towards lateralized stimuli [28,43,44]. Therefore, our N1_pc_-ERP component data suggests that orosensory signals may modulate initial attentional orienting processes, whereby CHO mouth rinsing seems to dampen attentional orienting to incongruent stimuli relative to NNS rinsing.

On the other hand, N2_pc_-ERP component amplitudes differed between congruent and incongruent trials in response to CHO rinsing. More specifically, larger N2_pc_-ERP component amplitudes were observed on congruent trials than incongruent ones. In contrast, no such effect was observed when the participants rinsed the NNS. Functionally, past studies have linked the N2_pc_-ERP component to attentional processes involved in target selection [29] and distractor suppression [30]. In terms of the functional interpretation of this study’s N2_pc_-ERP component, however, a particularity of our Simon task must be considered. In visual search tasks, though, typically used to investigate the N2_pc_-ERP component [12,45], a target stimulus must be identified among additional visual distractors. This, however, was not the case in our study. Instead, our cognitive task exclusively involved unilateral stimulus presentations, suggesting that suppression of additional visual distractor stimuli (besides the black background) was unnecessary. Therefore, given the nature of our cognitive task, we tentatively interpret the N2_pc_-ERP component as potentially reflecting a target selection process rather than distractor suppression. The N2_pc_-ERP component, in contrast to the N1_pc_-ERP component, further exhibited a main effect of congruency, which was characterized by larger amplitudes on congruent trials compared to incongruent trials overall.

Contrary to our brain data, mouth rinsing did not affect behavioral performance. More precisely, neither RTs nor %correct were affected by the type of solution rinsed. Of less relevance to this study’s research objective, we found congruency effects for both RTs and %correct. Thus, despite dealing with a Simon task programming error, our task was still effective in manipulating congruency as initially planned.

Considering our questionnaire data, mouth rinsing did affect ratings of sweetness and viscosity. Unfortunately, the CHO solution was rated sweeter and more viscous than the NNS solution. This finding illustrates unsuccessful taste matching, although we relied on taste-matched solutions from previous mouth-rinsing research [36]. This finding demonstrates unsuccessful taste matching between solutions, which we have discussed previously [15]. This issue may be traced back to aspartame’s (the NNS used in this study) instability within pH-neutral liquids. Therefore, we raise caution when using aspartame as a control condition in future mouth rinsing studies. While we observed an overall increase in stress and hunger over time, the opposite was obtained for affective valence. These changes are most likely due to the accumulation of fasting and task-induced stress. Regarding the increase in hunger, this effect was driven by the NNS condition. As previously speculated, CHO mouth rinsing may have counteracted hunger signals via, for example, cephalic mechanisms [15]. However, this interpretation remains purely speculative.

At this stage, it is crucial to address several fundamental questions. Firstly, do our results indicate the presence of ergogenic or ergolytic effects on neurocognition due to CHO mouth rinsing? Resolving this matter may prove to be a complex issue. Although we observed differences between solutions at the electrophysiological level, no such differences were apparent at the behavioral level. Therefore, mapping the observations made at both levels onto each other, which would have aided in interpreting the direction of our brain data, is a complex task. Moreover, most studies that have evaluated the electrophysiological correlates of visuospatial attention through Simon tasks have used bilateral target presentations instead of unilateral presentations [46]. This approach is typically used to prevent potential interference between ERP components linked to motor preparation and parietal–occipital attentional components [47]. Thus, the number of studies that can be referenced to facilitate interpreting our results is rather scarce.

Furthermore, linking our findings to previous research on the effects of CHO mouth rinsing on electrophysiological correlates of cognition is also severely limited. Specifically, to our knowledge, no other study has investigated the effects of CHO mouth rinsing on electrophysiological correlates associated with visuospatial attention. Although two studies examined the effects of CHO mouth rinsing on the P3-ERP component and interpreted it as an electrophysiological index of attention [13,14], their results can only contribute to the interpretation of our brain data to a limited extent. This is due to the inconsistent findings between the two studies, with one study [13] reporting ergogenic effects of NNS rinsing on P3-ERP component latency, while the other one [14] reported null results. Moreover, this is further complicated by the fact that the P3-ERP component is not functionally comparable to the N1_pc_ or N2_pc_-ERP component assessed in our study. In summary, the interpretation of our brain data is limited by the absence of significant effects of CHO mouth rinsing on overt behavior and the lack of comparable studies.

Thus, our interpretation of the study’s results can only be speculative at this stage. Nonetheless, we suggest that a slight shift in our understanding of how CHO mouth rinsing may influence both neurocognition (and possibly also physical performance) would enhance the interpretation of our findings and be of value for future research. Going beyond the prevailing belief that CHO mouth rinsing may improve (or worsen) specific functions or performance metrics, in general, may yield new insights. Therefore, we propose that our electrophysiological results may reflect the adoption of different “central control modes,” with neither mode proving superior in this study’s task context, as evidenced by the lack of differences in behavioral performance. Generally, depending on the specific task to be performed, whether cognitive or physical, shifting into a particular control mode may or may not yield adaptive benefits.

Applying this logic to our brain data, we propose that the modulated N1_pc_-ERP component amplitude we observed in response to CHO, relative to NNS, may reflect an attempt to reduce early attentional orienting towards incongruent targets to limit the processing of the conflicting spatial dimension. Thereby, the information processing system may ultimately aim to reduce conflict at later, more bottlenecked processing stages (e.g., response selection) [48]. In such a scenario, information processing may have been biased to detect incongruent stimuli as early as possible, thereby reducing attentional orienting toward incongruent stimuli. However, the means by which such early congruency discrimination may have occurred in response to CHO mouth rinsing remains elusive.

Compared to CHO mouth rinsing, the increased N1_pc_-ERP component amplitude observed during incongruent trials due to NNS rinsing could suggest that the information processing system responded similarly to each visual stimulus arrangement. In other words, the information processing system may have treated each trial equally, allocating attentional resources equally to congruent and incongruent trials. 

A potential reduction in early attentional processing of incongruent stimuli induced by CHO rinsing, however, could generate costs at later processing stages. More specifically, less attentional orienting to incongruent stimuli may have come at the expense of reductions in processing the relevant stimulus dimension (i.e., shape). Therefore, to achieve optimal behavioral performance, a need for compensation may arise at later processing stages. Our N2_pc_-ERP component results might indeed serve as an indicator of such speculation. The fact that N2_pc_-ERP component amplitude discriminated for congruency only in response to rinsing CHO could indicate that more attentional resources had to be allocated during target selection. In turn, under conditions of NNS rinsing, potentially stronger processing of the relevant stimulus dimension at an earlier orienting stage may lead to less pronounced attentional demands at later processing stages. In conclusion, we propose that CHO and NNS rinsing may have been associated with states of information processing biased to either more top-down or bottom-up processing, respectively.

If all the interpretations above remain speculative, hypotheses on how CHO mouth rinsing might induce shifts in control states mechanistically may motivate future research endeavors. While the exact mechanisms underlying the effects of CHO mouth rinsing remain unclear, imaging studies have indicated that CHO mouth rinsing may activate neural reward circuits stronger relative to NNS rinsing [7,8,21]. Building upon these findings, we have previously proposed that the 'liking' component of reward may play a crucial role in how CHO mouth rinsing affects cognition and physical performance [15]. More specifically, by dampening negative affective factors, such as error-induced negative affect [15], increases in positively valenced core affect may modulate information processing. Regarding the current study, it is highly probable that the implemented fasting manipulation triggered a considerable stress response. Interestingly, compelling evidence from cognitive neuroscience suggests that the activation of neural reward circuits can mitigate stress [49]. Specifically, these stress-buffering effects may primarily involve the 'liking' component of reward [49], which refers to the positively valenced core affect triggered by a pleasant sensory experience [11,24]. It is important to note that we are here referring to objective 'liking' as a neurobiological response, distinct from the subjective “liking” one experiences at the subjective level [24]. Considering this context, CHO mouth rinsing may have elicited a more robust 'liking' response than NNS rinsing, potentially providing greater buffering against the stress induced by fasting and task performance. NNS rinsing, in contrast, may have buffered overall stress in a minor way, possibly due to recruiting neural reward circuits to a lesser extent. As a result, it is conceivable that the information processing system was shifted from a state more strongly biased by sensory input toward a state of more top-down control. However, it is imperative to acknowledge that all the interpretations are entirely speculative. Hence, conducting further research to examine these assumptions systematically is crucial.

At this point, we would like to clarify all our study’s limitations. First, matching solutions concerning taste evaluation was unsuccessful (see [15]). As such, the NNS solution cannot be considered a placebo condition. Henceforth, we referred to it as NNS rinsing throughout the manuscript. Additionally, due to a programming error of the Simon task, counterbalancing of the response mapping was abolished and introduced an unanticipated between-subjects factor (fully detailed in the methods section). Moreover, we cannot guarantee that all participants adhered to the fasting period since it was not carried out under laboratory conditions, even though all participants provided written confirmation of their adherence to the fasting periods. Also, no blood glucose levels were assessed. Consequently, we cannot exclude that some glucose was peripherally available during CHO mouth rinsing. Lastly, due to economic considerations, we did not implement a neutral condition (such as water rinsing), which, in turn, led to the absence of a proper baseline condition. Therefore, we cannot infer the direction of our results with regard to a proper baseline.

## 5. Conclusions

In summary, we observed that fine-grained electrophysiological correlates of visuospatial attention differed depending on whether CHO or NNS availability was induced in the oral cavity using the mouth rinsing technique. Relative to rinsing an NNS solution, CHO mouth rinsing resulted in a decrease in earlier (more bottom-up controlled; N1_pc_-ERP component) and an increase in later (more top-down controlled; N2_pc_-ERP component) electrophysiological correlates of visuospatial attention; however, how these brain modulations reflect ergogenic or ergolytic properties of CHO mouth rinsing on neurocognition remains unclear. Based on pure speculation, CHO mouth rinsing, through affective modulations, might bias the information processing system toward more top-down control, which may or may not provide adaptive value depending on a specific environmental context. Regarding the transferability of our results to the often-presumed central mechanism underlying ergogenic effects of CHO mouth rinsing on endurance performance, our data might suggest the involvement of modulated attentional factors. However, this assumption is purely speculative and would require further research to test this hypothesis. From a methodological standpoint, however, our findings suggest the potential significance of employing neuroscientific techniques and manipulating nutritional states (e.g., inducing physiological appetite) to gain a more comprehensive understanding of the impact of CHO mouth rinsing on central-level processes.

## Data Availability

De-identified data can be provided by the corresponding author upon request.

## Supplementary Materials

The following supporting information can be downloaded at: https://www.mdpi.com/article/10.3390/nu15133053/s1.
