# Supplementary material for: How Attention Changes in Response to Carbohydrate Mouth Rinsing
Source: Nutrients. 2023 Jul 6;15(13):3053. doi: 10.3390/nu15133053 (PMC10346677; doi:10.3390/nu15133053)
Supplement: Supplementary file 1 [file nutrients-15-03053-s001.zip › nutrients-2438227-supplementary.pdf]

Experimental Conditions

Table S1. The sum of participants assigned to the experimental conditions.

utility	solution_seq	task_seq	n
low	nns_cho	flanker_simon	7
low	nns_cho	simon_flanker	5
low	cho_nns	flanker_simon	7
low	cho_nns	simon_flanker	7
high	nns_cho	flanker_simon	7
high	nns_cho	simon_flanker	7
high	cho_nns	flanker_simon	8
high	cho_nns	simon_flanker	5

Simon Task

A modified Simon task was used that consisted of 600 trials per session. Each trial started with presenting a centrally located fixation stimulus (shape: circle, RGB color code: [.5, .5, .5], presentation duration: randomly varying between 50 to 80 frames, degrees of visual angle: .05°). Subsequently, the fixation was joined by a lateralized (left or right hemifield, relative to fixation) target stimulus (shape: rectangular [horizontal or vertical orientation], RGB color code: [.5, .5, .5], presentation duration: 10 frames, degrees of visual angle: 2°). The response had to be given before a deadline initially set at 450 ms. This deadline was constantly updated relative to the proportions of correct responses in the preceding ten trials. On the one hand, if the proportions of correct responses were < 80 %, the deadline was increased by 50 ms. On the other hand, if the proportions of correct responses were above 90 %, the deadline was decreased by 50 ms. If the deadline was violated, feedback was provided in the form of the red-colored word "SCHNELLER" (faster). All stimuli were presented on a black background. Participants were asked to respond as fast and accurately as possible with their right or left index finger to the orientation of the rectangular. Stimulus-response mapping was instructed before the start of the task. Target stimulus position and orientation could therefore be congruent or incongruent in terms of the spatial location of the target stimulus and the spatial location of the required button press. Proportions of congruency were either 75% congruent vs. 25 % incongruent or 25 % congruent vs. 75% incongruent. Participants were assigned to either one of the congruency proportion manipulations. The Simon task was interrupted after every 150 trials to allow for performing a brief mouth rinse.

EEG Report

EEG amplifier:
Type: actiCHamp
Manufacturer: Brain Products GmbH (Gilching, Germany)

EEG recording software:
Type: BrainVision Recorder software
Version: v1.22.0001
Manufacturer: Brain Products GmbH (Gilching, Germany)

EEG sensor cap:
Type: actiCAP
Manufacturer: Brain Products GmbH (Gilching, Germany)

EEG sensor type: Ag/AgCl active electrodes

EEG sensor placement system: international 10-10 system

EEG sensor ids/locations: AF3, AF4, AF7, AF8, AFz, C1, C2, C3, C4, C5, C6, CP1, CP2, CP3, CP4, CP5, CP6, CPz, F1, F2, F3, F4, F5, F6, F7, F8, FC1, FC2, FC3, FC4, FC5, FC6, FCz, FT7, FT8, Fp1, Fp2, Fz, O1, O2, Oz, P1, P2, P3, P4, P5, P6, P7, P8, PO3, PO4, PO7, PO8, POz, Pz, TP10, TP7, TP8, TP9.

EOG sensors: Horizontal and vertical electrooculograms were measured with four EEG electrodes positioned at the left (EEG sensor FT9 --> LO1) and right outer canthi (EEG sensor FT10 --> LO2) and above (EEG sensor T8 --> SO2) and below the right eye (EEG sensor T7 --> IO2).

EEG recording sampling rate: 1000 Hz

EEG sensor impedances: < 10 kΩ

EEG recording ground sensor: Fpz

EEG recording reference sensor: Cz

EEG online filtering:
Type: IIR Butterworth 2nd order causal lowpass
l_freq: DC
h_freq: 280 Hz (-3 dB)

EEG offline processing sampling rate: 1000 Hz

Muscle artifacts: Muscle artifacts were automatically detected and annotated within the continuous data using the mne.preprocessing.annotate_muscle_zscore function. The 'threshold' parameter was set at five z-scores and the 'min_length_good' parameter was set at 0.2 s. Time periods containing any muscle artifact annotation were rejected during epoching.

Ocular artifacts: Ocular artifact correction was performed using independent component analysis using MNE's implementation of the picard algorithm. Therefore, ICA decomposition was run on a filtered (type: IIR Butterworth 4th order zero-phase highpass; lower frequency cutoff: 1 Hz (-6 dB)) and evenly time-segmented (1-sec) copy of the raw EEG data. Segments with large voltage deviations

(peak-to-peak deviations > {'eeg': 0.0005} µV in any EEG channel), non-task periods (pre-/post task & rinsing), as well as bad channels, were ignored during decomposition. Subsequently, MNE's 'find_bads_eog' function automatically detected independent components reflecting vertical or horizontal eye movements. This selection was then visually cross-checked (independently) by two experienced EEG researchers. Visual inspection was performed by comparing the timing and shape of the ICA components with the EOG signals and examining the components' topographies. In the case of no consensus, a third experienced EEG researcher was brought in to contribute to a final decision concerning component selection. This last component selection was then zeroed out from a filtered copy of the raw data (see EEG offline filtering).

EEG bad sensor detection: Bad EEG sensors were automatically detected via MNE implementation of the PREP pipeline (functions: "bad_by_nan", "bad_by_flat", "bad_by_deviation", "bad_by_hf_noise", "bad_by_correlation", "bad_by_SNR", "bad_by_dropout", "bad_by_ransac" (Bigdely-Shamlo et al., 2015 [40]).

EEG bad sensor interpolation: All bad EEG sensors were interpolated using spherical splines.

EEG offline filtering:

Notch filter:

Type: FIR one-pass, zero-phase, non-causal bandstop
Frequencies: 50, 100, 150, 200, 250
Filter length: 6601 samples

Bandpass filter:

Type: IIR Butterworth 4th order zero-phase bandpass
Lower frequency cutoff: 0.10 Hz (-6 dB)
Upper cutoff frequency: 30 Hz (-6 dB)

EEG data re-referencing: average reference

EEG data segmentation/epoching: The continuous data were segmented into target-locked epochs (correct trials) of 0.8 sec duration (0.2 sec pre-target interval; baseline correction interval: -0.2 : 0 sec).

EEG sensors selected for statistical analysis: All posterior and occipital electrodes (O1/2, P1/2, P3/4, P5/6, P7/8, PO3/4, PO7/8) were selected for the analysis of the N1pc and N2pc.

ERP component scoring:

The N1pc and N2pc were scored as mean amplitude. Component scoring ranges were identified unbiasedly using the "collapsed localizer technique" (Luck & Gaspelin 2017 [42]). Since the N2pc did not exhibit a clear peak, both components were scored as "peak-to-peak" to maintain scoring consistency between components. See Figure 1 for the collapsed localizer waveform used to identify the scoring time ranges.

N1pc scoring range = (0.89 s, 0.213 s)

N2pc scoring range = (0.213 s, 0.282 s)

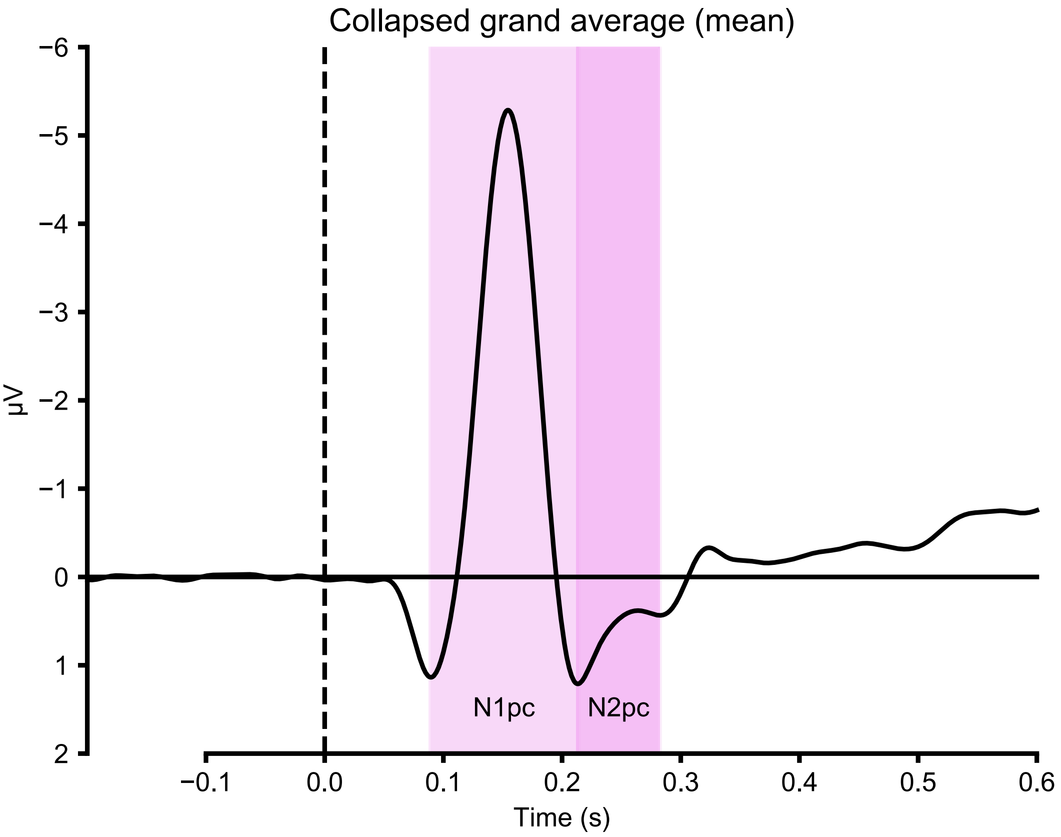

Figure S1. Grand average waveform for correct trials averaged across all conditions. The displayed data are re-referenced to the average reference and baseline corrected (−0.2 s : 0 s). The sections highlighted in purple represent the time intervals identified for ERP component scoring. Abbreviations: N1 posterior contralateral (N1pc), N2 posterior contralateral (N2pc).

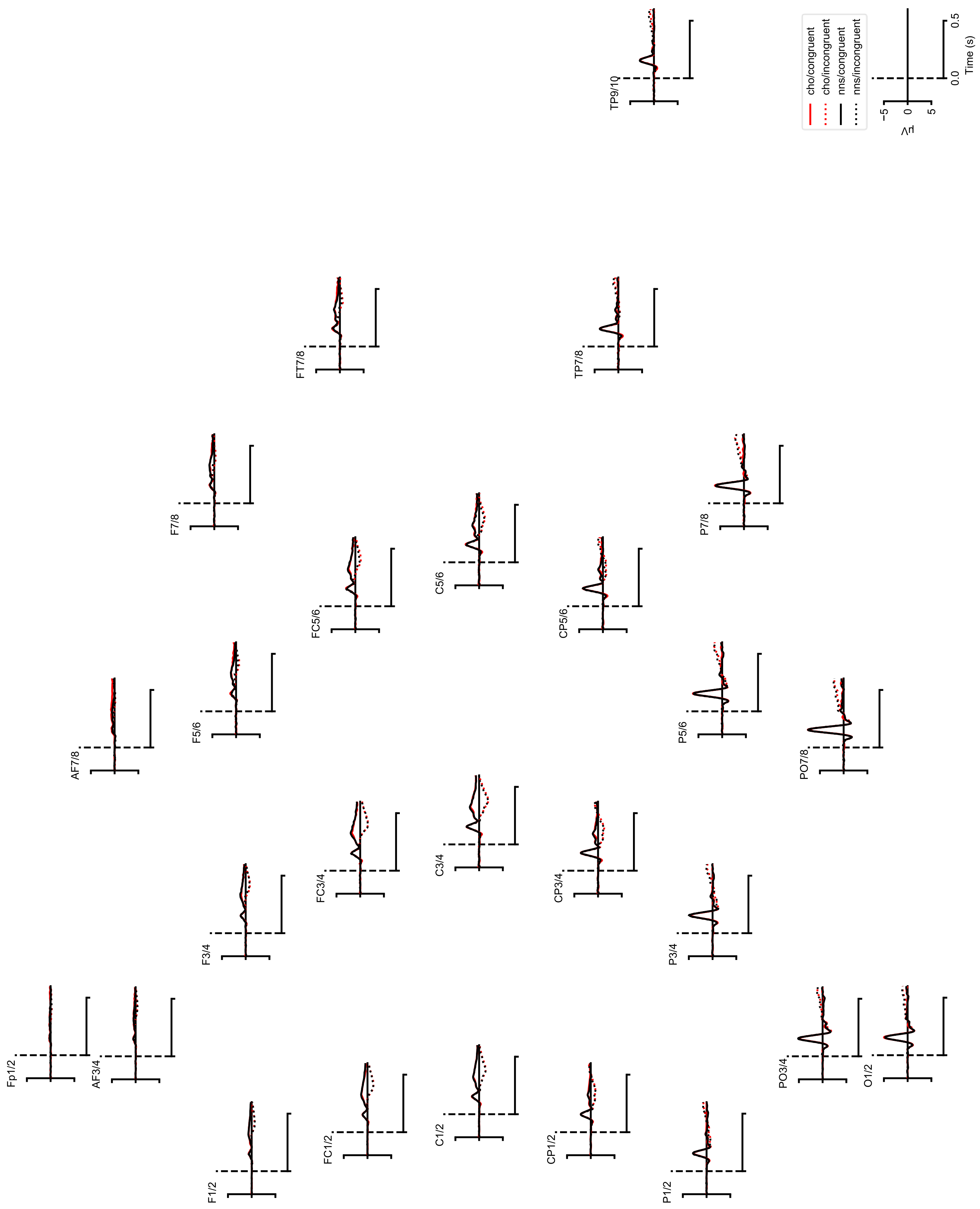

Figure S2. Grand average waveforms for the type of solution and congruency. Abbreviations: carbohydrate (cho), non-nutritive sweetener (nns).

Results

Preprocessing

Interpolated channels

Table S2. ANOVA Table for the number of interpolated channels.

dv_type	Cases	Sphericity_Correction	Mean_Squared_Error	df(num)	df(den)	F	p	partial_eta_squared
interpolated channels	solution	None	5.06	1	51	0.32	0.572	0.01
interpolated channels	utility	None	9.06	1	51	0.26	0.614	0.01
interpolated channels	solution: utility	None	5.06	1	51	0.16	0.691	0

Table S3. Descriptive statistics for number of interpolated channels.

dv_type	solution	utility	N	Mean	SD	SE
interpolated channels	cho	high	26	4.731	3.584	0.703
interpolated channels	cho	low	27	4.259	2.086	0.402
interpolated channels	nns	high	26	4.308	2.71	0.531
interpolated channels	nns	low	27	4.185	1.981	0.381

Removed ocular ICA components

Table S4. ANOVA Table for removed ocular components.

dv_type	Cases	Sphericity_Correction	Mean_Squared_Error	df(num)	df(den)	F	p	partial_eta_squared
icaComponent	solution	None	0.13	1	51	0.68	0.412	0.01
icaComponent	utility	None	0.25	1	51	1.08	0.304	0.02
icaComponent	solution:utility	None	0.13	1	51	0.08	0.773	0

Table S5. Descriptive statistics for removed ocular components.

dv_type	solution	utility	N	Mean	SD	SE
icaComponent	cho	high	26	1.731	0.604	0.118
icaComponent	cho	low	27	1.852	0.362	0.07
icaComponent	nns	high	26	1.808	0.402	0.079
icaComponent	nns	low	27	1.889	0.32	0.062

Trials included into analysis

Table S6. ANOVA Table for trials included into analysis.

dv_type	Cases	Sphericity_Correction	Mean_Squared_Error	df(num)	df(den)	F	p	partial_eta_squared
trials	congruency	None	295.87	1	51	70.02	0	0.58
trials	solution	None	434.73	1	51	0.39	0.536	0.01
trials	utility	None	664.55	1	51	8.33	0.006	0.14
trials	congruency:utility	None	295.87	1	51	8751.46	0	0.99
trials	solution:utility	None	434.73	1	51	0.03	0.866	0
trials	solution:congruency	None	102.96	1	51	0.8	0.375	0.02
trials	congruency:solution:utility	None	102.96	1	51	0.58	0.45	0.01

Table S7. Descriptive statistics for the trials included into analysis.

dv_type	solution	utility	congruency	N	Mean	SD	SE
trials	cho	high	congruent	26	326.615	19.079	3.742
trials	cho	high	incongruent	26	83.462	13.846	2.715
trials	cho	low	congruent	27	93.778	9.399	1.809
trials	cho	low	incongruent	27	294.889	25.906	4.986
trials	nns	high	congruent	26	322.038	22.238	4.361
trials	nns	high	incongruent	26	83.5	11.158	2.188
trials	nns	low	congruent	27	92.296	12.316	2.37
trials	nns	low	incongruent	27	293.778	29.966	5.767

Table S8. Post Hoc Comparisons—CONGRUENCY.

dv_type	contrast	estimate	SE	df	t.ratio	p.value	cohens_d
trials	congruent-incongruent	19.8	2.36	51	8.368	0	1.02

Table S9. Post Hoc Comparisons—UTILITY.

dv_type	contrast	estimate	SE	df	t.ratio	p.value	cohens_d
trials	high-low	10.2	3.54	51	2.885	0.0057	0.528

Table S10. Post Hoc Comparisons—CONGRUENCY x UTILITY.

dv_type	contrast	estimate	SE	df	t.ratio	p.value	cohens_d
trials	high_congruent-low_congruent	231.29	3.51	51	65.825	0	11.951
trials	high_congruent-high_incongruent	240.85	3.37	51	71.396	0	12.445
trials	high_congruent-low_incongruent	29.99	4.24	51	7.066	0	1.55
trials	low_congruent-high_incongruent	9.56	4.27	51	2.238	0.0296	0.494
trials	low_congruent-low_incongruent	-201.3	3.31	51	-60.809	0	-10.401
trials	high_incongruent-low_incongruent	-210.85	4.89	51	-43.122	0	-10.895

Table S11. Trials included into analysis for each participant.

dv_type	participant	utility	nns_congruent	nns_incongruent	cho_congruent	cho_incongruent
trials	1	high	338	90	319	87
trials	2	high	322	83	296	62
trials	3	low	76	251	90	262
trials	4	low	87	275	101	330
trials	5	low	90	303	99	325
trials	6	high	301	74	314	90
trials	7	low	108	333	84	310
trials	8	low	62	242	91	300
trials	9	high	362	101	321	91
trials	10	low	104	318	95	296
trials	11	high	362	86	362	105
trials	12	high	344	100	318	87
trials	13	high	310	87	323	87
trials	14	low	87	274	88	289
trials	15	high	330	80	306	58
trials	16	high	333	95	321	94
trials	17	low	88	272	89	286
trials	18	high	298	66	313	67
trials	20	low	105	323	91	293
trials	22	high	291	75	317	69
trials	25	high	363	100	328	77
trials	26	low	80	233	92	243
trials	27	low	103	303	106	317
trials	28	high	290	89	330	94
trials	29	low	80	293	88	285
trials	30	low	91	310	77	240
trials	31	high	308	79	312	82
trials	33	high	315	83	329	93

trials	34	high	317	81	350	93
trials	35	low	110	316	105	310
trials	37	low	74	225	111	282
trials	38	high	320	94	336	77
trials	39	low	88	299	101	341
trials	40	high	308	91	330	104
trials	41	high	293	59	288	56
trials	42	high	347	91	329	83
trials	43	low	104	277	100	288
trials	44	low	104	332	98	280
trials	45	high	313	86	352	101
trials	47	high	297	79	348	89
trials	48	low	81	309	82	303
trials	49	high	326	92	327	67
trials	51	low	109	337	83	297
trials	52	high	332	64	370	98
trials	53	low	109	296	83	283
trials	54	low	96	292	100	286
trials	55	low	91	291	103	297
trials	56	low	90	297	80	283
trials	57	high	344	71	342	73
trials	58	low	94	322	86	289
trials	59	high	309	75	311	86
trials	60	low	85	318	110	360
trials	61	low	96	291	99	287

EEG

N1pc

Table S12. ANOVA Table for N1pc amplitude.

dv_type	Cases	Sphericity_Correction	Mean_Squared_Error	df(num)	df(den)	F	p	partial_eta_squared
n1pc	congruency	None	0.17	1	51	0.15	0.704	0
n1pc	solution	None	0.15	1	51	3.05	0.087	0.06
n1pc	utility	None	5.21	1	51	0.15	0.703	0
n1pc	congruency:utility	None	0.17	1	51	4.25	0.044	0.08
n1pc	solution:utility	None	0.15	1	51	0.01	0.913	0
n1pc	solution:congruency	None	0.04	1	51	9.04	0.004	0.15
n1pc	congruency:solution:utility	None	0.04	1	51	0.02	0.879	0

Table S13. Descriptive statistics for N1pc amplitude.

dv_type	solution	utility	congruency	N	Mean	SD	SE
n1pc	cho	high	congruent	26	-1.69	1.1	0.216
n1pc	cho	high	incongruent	26	-1.701	1.012	0.198
n1pc	cho	low	congruent	27	-1.918	1.237	0.238
n1pc	cho	low	incongruent	27	-1.701	1.068	0.206
n1pc	nns	high	congruent	26	-1.693	1.181	0.232
n1pc	nns	high	incongruent	26	-1.874	1.235	0.242
n1pc	nns	low	congruent	27	-1.941	1.295	0.249
n1pc	nns	low	incongruent	27	-1.878	1.275	0.245

Table S14. Post Hoc Comparisons—CONGRUENCY x UTILITY.

dv_type	contrast	estimate	SE	df	t.ratio	p.value	cohens_d
n1pc	low_congruent-low_incongruent	-0.13993	0.0802	51	-1.744	0.3486	-0.1185
n1pc	high_congruent-high_incongruent	0.09624	0.0818	51	1.177	0.7338	0.0815
n1pc	low_congruent-high_congruent	-0.23829	0.3262	51	-0.730	0.9370	-0.2019
n1pc	low_incongruent-high_incongruent	-0.00212	0.3111	51	-0.007	0.9946	-0.0018

Table S15. Post Hoc Comparisons—SOLUTION x CONGRUENCY.

dv_type	contrast	estimate	SE	df	t.ratio	p.value	cohens_d
n1pc	nns_congruent-nns_incongruent	0.0591	0.0703	51	0.841	0.8090	0.0501
n1pc	cho_congruent-cho_incongruent	-0.1028	0.0554	51	-1.856	0.2075	-0.0871
n1pc	nns_congruent-cho_congruent	-0.0127	0.0594	51	-0.214	0.8310	-0.0108
n1pc	nns_incongruent-cho_incongruent	-0.1747	0.0606	51	-2.884	0.0230	-0.1480

N2pc

Table S16. ANOVA Table for N2pc amplitude.

dv_type	Cases	Sphericity_Correction	Mean_Squared_Error	df(num)	df(den)	F	p	partial_eta_squared
n2pc	congruency	None	0.44	1	51	15.05	0	0.23
n2pc	solution	None	0.22	1	51	0.34	0.56	0.01
n2pc	utility	None	3.35	1	51	2.3	0.136	0.04
n2pc	congruency:utility	None	0.44	1	51	20.71	0	0.29
n2pc	solution:utility	None	0.22	1	51	1.18	0.283	0.02
n2pc	solution:congruency	None	0.16	1	51	5.88	0.019	0.1
n2pc	congruency:solution:utility	None	0.16	1	51	0.51	0.477	0.01

Table S17. Descriptive statistics for N2pc amplitude.

dv_type	solution	utility	congruency	N	Mean	SD	SE
n2pc	cho	high	congruent	26	0.818	1.007	0.198
n2pc	cho	high	incongruent	26	0.851	0.922	0.181
n2pc	cho	low	congruent	27	-0.089	1.059	0.204
n2pc	cho	low	incongruent	27	0.855	1.023	0.197
n2pc	nns	high	congruent	26	0.88	0.977	0.192
n2pc	nns	high	incongruent	26	0.724	0.909	0.178
n2pc	nns	low	congruent	27	0.192	1.004	0.193
n2pc	nns	low	incongruent	27	0.789	1.229	0.237

Table S18. Post Hoc Comparisons—CONGRUENCY.

dv_type	contrast	estimate	SE	df	t.ratio	p.value	cohens_d
n2pc	congruent-incongruent	-0.354	0.0913	51	-3.879	0.0003	-0.347

Table S19. Post Hoc Comparisons—CONGRUENCY x UTILITY.

dv_type	contrast	estimate	SE	df	t.ratio	p.value	cohens_d
n2pc	low_congruent-low_incongruent	-0.7700	0.128	51	-6.018	<.0001	-0.7537
n2pc	high_congruent-high_incongruent	0.0614	0.130	51	0.471	1.0000	0.0601
n2pc	low_congruent-high_congruent	-0.7971	0.267	51	-2.984	0.0131	-0.7802
n2pc	low_incongruent-high_incongruent	0.0343	0.268	51	0.128	1.0000	0.0336

Table S20. Post Hoc Comparisons—SOLUTION x CONGRUENCY.

dv_type	contrast	estimate	SE	df	t.ratio	p.value	cohens_d
n2pc	nns_congruent-nns_incongruent	-0.221	0.1113	51	-1.983	0.1055	-0.2159
n2pc	cho_congruent-cho_incongruent	-0.488	0.1019	51	-4.789	0.0001	-0.4776
n2pc	nns_congruent-cho_congruent	0.171	0.0778	51	2.204	0.0962	0.1677
n2pc	nns_incongruent-cho_incongruent	-0.096	0.0910	51	-1.054	0.2968	-0.0939

Behavior

Response times (rt)

Table S21. ANOVA Table for response times.

dv_type	Cases	Sphericity_Correction	Mean_Squared_Error	df(num)	df(den)	F	p	partial_eta_squared
rt	congruency	None	312.28	1	51	193.43	0	0.79
rt	solution	None	1578.4	1	51	0.17	0.682	0
rt	utility	None	9528.45	1	51	4.89	0.031	0.09
rt	congruency:utility	None	312.28	1	51	232.35	0	0.82
rt	solution:utility	None	1578.4	1	51	1.5	0.226	0.03
rt	solution:congruency	None	110.41	1	51	1.59	0.214	0.03
rt	congruency:solution:utility	None	110.41	1	51	1.3	0.26	0.02

Table S22. Descriptive statistics for response times.

dv_type	solution	utility	congruency	N	Mean	SD	SE
rt	cho	high	congruent	26	374.805	37.825	7.418
rt	cho	high	incongruent	26	445.75	49.357	9.68
rt	cho	low	congruent	27	387.185	61.506	11.837
rt	cho	low	incongruent	27	387.407	60.563	11.655
rt	nns	high	congruent	26	379.41	46.198	9.06
rt	nns	high	incongruent	26	450.01	58.514	11.476
rt	nns	low	congruent	27	381.713	59.318	11.416
rt	nns	low	incongruent	27	375.007	50.829	9.782

Table S23. Post Hoc Comparisons—CONGREUNCY.

dv_type	contrast	estimate	SE	df	t.ratio	p.value	cohens_d
rt	congruent-incongruent	-33.8	2.43	51	-13.908	<.0001	-0.629

Table S24. Post Hoc Comparisons—UTILITY.

dv_type	contrast	estimate	SE	df	t.ratio	p.value	cohens_d
rt	high-low	29.7	13.4	51	2.212	0.0315	0.553

Table S25. Post Hoc Comparisons—CONGRUENCY x UTILITY.

dv_type	contrast	estimate	SE	df	t.ratio	p.value	cohens_d
rt	low_congruent-low_incongruent	3.24	3.40	51	0.953	0.6898	0.0604
rt	high_congruent-high_incongruent	-70.77	3.47	51	-20.421	<.0001	-1.3182
rt	low_congruent-high_congruent	7.34	13.38	51	0.549	0.6898	0.1367
rt	low_incongruent-high_incongruent	-66.67	13.87	51	-4.807	<.0001	-1.2419

Proportions of correct responses (%correct)

Table S26. ANOVA Table for proportions of correct responses.

dv_type	Cases	Sphericity_Correction	Mean_Squared_Error	df(num)	df(den)	F	p	partial_eta_squared
%correct	congruency	None	58.48	1	51	79.32	0	0.61
%correct	solution	None	24.73	1	51	0.54	0.467	0.01
%correct	utility	None	49.35	1	51	3.43	0.07	0.06
%correct	congruency:utility	None	58.48	1	51	215.3	0	0.81
%correct	solution:utility	None	24.73	1	51	3.29	0.076	0.06
%correct	solution:congruency	None	18.71	1	51	1.11	0.296	0.02
%correct	congruency:solution:utility	None	18.71	1	51	0.03	0.855	0

Table S27. Descriptive statistics for proportions of correct responses.

dv_type	solution	utility	congruency	N	Mean	SD	SE
%correct	cho	high	congruent	26	96.576	1.888	0.37
%correct	cho	high	incongruent	26	71.285	10.31	2.022
%correct	cho	low	congruent	27	84.295	6.599	1.27
%correct	cho	low	incongruent	27	89.618	3.64	0.7
%correct	nns	high	congruent	26	96.797	1.859	0.365
%correct	nns	high	incongruent	26	72.543	8.341	1.636
%correct	nns	low	congruent	27	81.819	6.693	1.288
%correct	nns	low	incongruent	27	88.614	4.392	0.845

Table S28. Post Hoc Comparisons—CONGRUENCY.

dv_type	contrast	estimate	SE	df	t.ratio	p.value	cohens_d
%correct	congruent-incongruent	9.36	1.05	51	8.906	<.0001	1.52

Table S29. Post Hoc Comparisons—CONGRUENCY x UTILITY.

dv_type	contrast	estimate	SE	df	t.ratio	p.value	cohens_d
%correct	low_congruent-low_incongruent	-6.06	1.47	51	-4.117	0.0001	-0.985
%correct	high_congruent-high_incongruent	24.77	1.50	51	16.518	<.0001	4.028
%correct	low_congruent-high_congruent	-13.63	1.04	51	-13.134	<.0001	-2.216
%correct	low_incongruent-high_incongruent	17.20	1.73	51	9.942	<.0001	2.797

Questionnaires

Hunger

Table S30. ANOVA Table for hunger ratings.

dv_type	Cases	Sphericity_Correction	Mean_Squared_Error	df(num)	df(den)	F	p	partial_eta_squared
hunger	solution	None	5.7	1	51	0.2	0.655	0
hunger	tp	None	2.92	2	102	9.77	0	0.16
hunger	utility	None	36.73	1	51	0.04	0.849	0
hunger	solution:utility	None	5.7	1	51	1.2	0.279	0.02
hunger	solution:tp	Greenhouse-Geisser	3.03	1.59	80.95	4.6	0.019	0.08
hunger	tp:utility	None	2.92	2	102	1.84	0.165	0.03
hunger	solution:tp:utility	Greenhouse-Geisser	3.03	1.59	80.95	0.38	0.634	0.01

Table S31. Descriptive statistics for hunger ratings.

dv_type	solution	utility	tp	N	Mean	SD	SE
hunger	cho	high	tp1	27	5.56	2.684	0.517
hunger	cho	high	tp2	27	5.411	3.302	0.636
hunger	cho	high	tp3	27	6.056	3.406	0.655
hunger	cho	low	tp1	26	5.95	2.744	0.538
hunger	cho	low	tp2	26	5.083	2.818	0.553
hunger	cho	low	tp3	26	5.505	3.159	0.619
hunger	nns	high	tp1	27	4.796	3.122	0.601
hunger	nns	high	tp2	27	4.529	3.181	0.612
hunger	nns	high	tp3	27	6.461	3.135	0.603
hunger	nns	low	tp1	26	5.512	2.696	0.529
hunger	nns	low	tp2	26	5.215	2.508	0.492
hunger	nns	low	tp3	26	6.328	2.736	0.537

Table S32. Post Hoc Comparisons—SOLUTION x TP.

dv_type	contrast	estimate	SE	df	t.ratio	p.value	cohens_d
hunger	nns_tp1-nns_tp2	0.2814	0.246	51	1.146	0.7548	0.09462
hunger	nns_tp1-nns_tp3	-1.2409	0.289	51	-4.299	0.0006	-0.41722
hunger	nns_tp2-nns_tp3	-1.5223	0.330	51	-4.615	0.0002	-0.51183
hunger	cho_tp1-cho_tp2	0.5081	0.282	51	1.799	0.4679	0.17083

hunger	cho_tp1-cho_tp3	-0.0256	0.406	51	-0.063	0.9498	-0.00862
hunger	cho_tp2-cho_tp3	-0.5337	0.326	51	-1.640	0.5361	-0.17946
hunger	nns_tp1-cho_tp1	-0.6011	0.306	51	-1.964	0.3848	-0.20209
hunger	nns_tp2-cho_tp2	-0.3744	0.301	51	-1.243	0.7548	-0.12588
hunger	nns_tp3-cho_tp3	0.6142	0.461	51	1.332	0.7548	0.20650

Table S33. Post Hoc Comparisons—TP.

dv_type	contrast	estimate	SE	df	t.ratio	p.value	cohens_d
hunger	tp1-tp2	0.395	0.218	51	1.808	0.0766	0.133
hunger	tp1-tp3	-0.633	0.262	51	-2.414	0.0389	-0.213
hunger	tp2-tp3	-1.028	0.220	51	-4.664	0.0001	-0.346

Stress

Table S34. ANOVA Table for stress ratings.

dv_type	Cases	Sphericity_Correction	Mean_Squared_Error	df(num)	df(den)	F	p	partial_eta_squared
stress	solution	None	2.06	1	51	0.15	0.7	0
stress	tp	Greenhouse-Geisser	3.74	1.51	76.86	14.17	0	0.22
stress	utility	None	11.25	1	51	4.91	0.031	0.09
stress	solution:utility	None	2.06	1	51	1.3	0.26	0.02
stress	solution:tp	Greenhouse-Geisser	1.81	1.64	83.58	0.69	0.476	0.01
stress	tp:utility	Greenhouse-Geisser	3.74	1.51	76.86	4.57	0.022	0.08
stress	solution:tp:utility	Greenhouse-Geisser	1.81	1.64	83.58	0.66	0.492	0.01

Table S35. Descriptive statistics for stress ratings.

dv_type	solution	utility	tp	N	Mean	SD	SE
stress	cho	high	tp1	27	1.708	1.724	0.332
stress	cho	high	tp2	27	1.601	1.531	0.295
stress	cho	high	tp3	27	2.251	2.441	0.47
stress	cho	low	tp1	26	2.25	1.775	0.348
stress	cho	low	tp2	26	1.767	1.944	0.381
stress	cho	low	tp3	26	3.493	2.453	0.481
stress	nns	high	tp1	27	1.433	1.414	0.272
stress	nns	high	tp2	27	1.561	1.422	0.274
stress	nns	high	tp3	27	1.828	1.781	0.343
stress	nns	low	tp1	26	1.984	1.552	0.304
stress	nns	low	tp2	26	2.048	1.453	0.285
stress	nns	low	tp3	26	3.842	2.831	0.555

Table S36. Post Hoc Comparisons—UTILITY.

dv_type	contrast	estimate	SE	df	t.ratio	p.value	cohens_d
stress	high-low	-0.834	0.376	51	-2.216	0.0312	-0.436

Table S37. Post Hoc Comparisons—UTILITY x TP.

dv_type	contrast	estimate	SE	df	t.ratio	p.value	cohens_d
stress	low_tp1-low_tp2	0.2098	0.217	51	0.969	1.0000	0.10980
stress	low_tp1-low_tp3	-1.5504	0.382	51	-4.055	0.0014	-0.81134

stress	low_tp2-low_tp3	-1.7602	0.363	51	-4.848	0.0001	-0.92114
stress	high_tp1-high_tp2	-0.0109	0.213	51	-0.051	1.0000	-0.00572
stress	high_tp1-high_tp3	-0.4692	0.375	51	-1.250	1.0000	-0.24553
stress	high_tp2-high_tp3	-0.4582	0.356	51	-1.286	1.0000	-0.23981
stress	low_tp1-high_tp1	0.5467	0.396	51	1.380	1.0000	0.28612
stress	low_tp2-high_tp2	0.3260	0.387	51	0.842	1.0000	0.17060
stress	low_tp3-high_tp3	1.6279	0.575	51	2.832	0.0462	0.85193

Table S38. Post Hoc Comparisons—TP.

**dv_type**	**contrast**	**estimate**	**SE**	**df**	**t.ratio**	**p.value**	**cohens_d**
stress	tp1-tp2	0.0994	0.152	51	0.655	0.5153	0.052
stress	tp1-tp3	-1.0098	0.268	51	-3.770	0.0009	-0.528
stress	tp2-tp3	-1.1092	0.254	51	-4.361	0.0002	-0.580

Arousal

Table S39. ANOVA Table for arousal ratings.

dv_type	Cases	Sphericity_Correction	Mean_Squared_Error	df(num)	df(den)	F	p	partial_eta_squared
arousal	solution	None	2.32	1	51	2.4	0.127	0.04
arousal	tp	Greenhouse-Geisser	2.43	1.52	77.4	0.35	0.644	0.01
arousal	utility	None	7.11	1	51	6.08	0.017	0.11
arousal	solution:utility	None	2.32	1	51	0.13	0.716	0
arousal	solution:tp	Greenhouse-Geisser	1.09	1.51	76.97	2.25	0.125	0.04
arousal	tp:utility	Greenhouse-Geisser	2.43	1.52	77.4	0.68	0.47	0.01
arousal	solution:tp:utility	Greenhouse-Geisser	1.09	1.51	76.97	0.44	0.587	0.01

Table S40. Descriptive statistics for arousal ratings.

dv_type	solution	utility	tp	N	Mean	SD	SE
arousal	cho	high	tp1	27	2.685	1.449	0.279
arousal	cho	high	tp2	27	2.655	1.374	0.264
arousal	cho	high	tp3	27	2.519	1.221	0.235
arousal	cho	low	tp1	26	3.308	1.379	0.27
arousal	cho	low	tp2	26	3.038	1.399	0.274
arousal	cho	low	tp3	26	3.538	2.005	0.393
arousal	nns	high	tp1	27	2.667	1.359	0.261
arousal	nns	high	tp2	27	3	1.414	0.272
arousal	nns	high	tp3	27	2.799	1.469	0.283
arousal	nns	low	tp1	26	3.346	1.548	0.304
arousal	nns	low	tp2	26	3.769	1.986	0.39
arousal	nns	low	tp3	26	3.75	1.986	0.39

Table S41. Post Hoc Comparisons—UTILITY.

dv_type	contrast	estimate	SE	df	t.ratio	p.value	cohens_d
arousal	high-low	-0.738	0.299	51	-2.466	0.0170	-0.47

Affective valence

Table S42. ANOVA Table for affective valence ratings.

dv_type	Cases	Sphericity_Correction	Mean_Squared_Error	df(num)	df(den)	F	p	partial_eta_squared
affective valence	solution	None	2.56	1	51	0.22	0.641	0
affective valence	tp	Greenhouse-Geisser	1.38	1.77	90.13	13.02	0	0.2
affective valence	utility	None	9.83	1	51	1.45	0.234	0.03
affective valence	solution:utility	None	2.56	1	51	0.06	0.8	0
affective valence	solution:tp	Greenhouse-Geisser	0.99	1.76	89.86	1.56	0.217	0.03
affective valence	tp:utility	Greenhouse-Geisser	1.38	1.77	90.13	4.35	0.02	0.08
affective valence	solution:tp:utility	Greenhouse-Geisser	0.99	1.76	89.86	0.23	0.765	0

Table S43. Descriptive statistics for affective valence.

dv_type	solution	utility	tp	N	Mean	SD	SE
affective valence	cho	high	tp1	27	6.63	1.668	0.321
affective valence	cho	high	tp2	27	7.009	1.291	0.248
affective valence	cho	high	tp3	27	6.5	1.803	0.347
affective valence	cho	low	tp1	26	6.654	1.81	0.355
affective valence	cho	low	tp2	26	6.615	1.675	0.329
affective valence	cho	low	tp3	26	5.462	1.86	0.365
affective valence	nns	high	tp1	27	6.741	1.403	0.27
affective valence	nns	high	tp2	27	6.63	1.69	0.325
affective valence	nns	high	tp3	27	6.379	1.643	0.316
affective valence	nns	low	tp1	26	6.692	1.594	0.313
affective valence	nns	low	tp2	26	6.308	1.644	0.322
affective valence	nns	low	tp3	26	5.615	1.791	0.351

Table S44. Post Hoc Comparisons—TP x UTILITY.

dv_type	contrast	estimate	SE	df	t.ratio	p.value	cohens_d
affective valence	low_tp1-low_tp2	0.2115	0.174	51	1.217	1.0000	0.12726
affective valence	low_tp1-low_tp3	1.1346	0.230	51	4.923	0.0001	0.68258
affective valence	low_tp2-low_tp3	0.9231	0.241	51	3.837	0.0028	0.55532
affective valence	high_tp1-high_tp2	-0.1343	0.171	51	-0.787	1.0000	-0.08077
affective valence	high_tp1-high_tp3	0.2457	0.226	51	1.087	1.0000	0.14784
affective valence	high_tp2-high_tp3	0.3800	0.236	51	1.610	0.6818	0.22861
affective valence	low_tp1-high_tp1	-0.0121	0.367	51	-0.033	1.0000	-0.00728
affective valence	low_tp2-high_tp2	-0.3579	0.367	51	-0.974	1.0000	-0.21531
affective valence	low_tp3-high_tp3	-0.9010	0.440	51	-2.046	0.3212	-0.54203

Table S45. Post Hoc Comparisons—TP.

dv_type	contrast	estimate	SE	df	t.ratio	p.value	cohens_d
affective valence	tp1-tp2	0.0386	0.122	51	0.317	0.7523	0.0232
affective valence	tp1-tp3	0.6902	0.161	51	4.275	0.0003	0.4152
affective valence	tp2-tp3	0.6515	0.169	51	3.866	0.0006	0.3920

Sweetness

Table S46. ANOVA Table for sweetness ratings.

dv_type	Cases	Sphericity_Correction	Mean_Squared_Error	df(num)	df(den)	F	p	partial_eta_squared
sweetness	solution	None	6.74	1	51	48.54	0	0.49
sweetness	tp	None	0.98	1	51	1.16	0.286	0.02
sweetness	utility	None	8.25	1	51	1.91	0.173	0.04
sweetness	solution:utility	None	6.74	1	51	1.25	0.269	0.02
sweetness	solution:tp	None	1.17	1	51	1.02	0.318	0.02
sweetness	tp:utility	None	0.98	1	51	1.97	0.167	0.04
sweetness	solution:tp:utility	None	1.17	1	51	0.11	0.744	0

Table S47. Descriptive statistics for sweetness ratings.

dv_type	solution	utility	tp	N	Mean	SD	SE
sweetness	cho	high	tp2	27	7.767	1.534	0.295
sweetness	cho	high	tp3	27	7.71	1.287	0.248
sweetness	cho	low	tp2	26	8.153	1.275	0.25
sweetness	cho	low	tp3	26	7.618	1.627	0.319
sweetness	nns	high	tp2	27	4.781	2.722	0.524
sweetness	nns	high	tp3	27	4.927	2.511	0.483
sweetness	nns	low	tp2	26	5.867	2.407	0.472
sweetness	nns	low	tp3	26	5.729	2.542	0.498

Table S48. Post Hoc Comparisons—SOLUTION.

dv_type	contrast	estimate	SE	df	t.ratio	p.value	cohens_d
sweetness	cho-nns	2.49	0.357	51	6.967	<.0001	1.2

Viscosity

Table S49. ANOVA Table for affective viscosity ratings.

dv_type	Cases	Sphericity_Correction	Mean_Squared_Error	df(num)	df(den)	F	p	partial_eta_squared
viscosity	solution	None	2.3	1	51	9.44	0.003	0.16
viscosity	tp	None	0.72	1	51	0.49	0.486	0.01
viscosity	utility	None	9.29	1	51	2.28	0.137	0.04
viscosity	solution:utility	None	2.3	1	51	0.01	0.908	0
viscosity	solution:tp	None	0.64	1	51	1.24	0.271	0.02
viscosity	tp:utility	None	0.72	1	51	0	0.983	0
viscosity	solution:tp:utility	None	0.64	1	51	0.14	0.706	0

Table S50. Descriptive statistics for viscosity ratings.

dv_type	solution	utility	tp	N	Mean	SD	SE
viscosity	cho	high	tp2	27	8.033	2.423	0.466
viscosity	cho	high	tp3	27	8.034	1.857	0.357
viscosity	cho	low	tp2	26	7.338	1.91	0.375
viscosity	cho	low	tp3	26	7.417	1.702	0.334
viscosity	nns	high	tp2	27	8.73	1.44	0.277
viscosity	nns	high	tp3	27	8.57	1.58	0.304
viscosity	nns	low	tp2	26	8.166	1.555	0.305
viscosity	nns	low	tp3	26	7.918	1.73	0.339

Table S51. Post Hoc Comparisons—SOLUTION.

dv_type	contrast	estimate	SE	df	t.ratio	p.value	cohens_d
viscosity	cho-nns	-0.641	0.209	51	-3.072	0.0034	-0.356

Liking

Table S52. ANOVA Table for liking ratings.

dv_type	Cases	Sphericity_Correction	Mean_Squared_Error	df(num)	df(den)	F	p	partial_eta_squared
liking	solution	None	4.26	1	51	0.31	0.581	0.01
liking	tp	None	1.76	1	51	0	0.95	0
liking	utility	None	15.79	1	51	0.5	0.483	0.01
liking	solution:utility	None	4.26	1	51	0.26	0.612	0.01
liking	solution:tp	None	1.08	1	51	0.28	0.597	0.01
liking	tp:utility	None	1.76	1	51	2.34	0.132	0.04
liking	solution:tp:utility	None	1.08	1	51	2.32	0.134	0.04

Table S53. Descriptive statistics for liking ratings.

dv_type	solution	utility	tp	N	Mean	SD	SE
liking	cho	high	tp2	27	4.267	2.595	0.499
liking	cho	high	tp3	27	4.828	2.727	0.525
liking	cho	low	tp2	26	5.005	2.441	0.479
liking	cho	low	tp3	26	4.573	2.375	0.466
liking	nns	high	tp2	27	4.258	1.889	0.364
liking	nns	high	tp3	27	4.232	2.27	0.437
liking	nns	low	tp2	26	4.851	1.991	0.39
liking	nns	low	tp3	26	4.701	2.704	0.53

Task difficulty

Table S54. ANOVA Table for task difficulty ratings.

dv_type	Cases	Sphericity_Correction	Mean_Squared_Error	df(num)	df(den)	F	p	partial_eta_squared
difficulty	solution	None	4.63	1	51	3.34	0.073	0.06
difficulty	utility	None	9.24	1	51	0.29	0.592	0.01
difficulty	solution:utility	None	4.63	1	51	0.49	0.489	0.01

Table S55. Descriptive statistics for task difficulty ratings.

dv_type	solution	utility	N	Mean	SD	SE
difficulty	cho	high	27	4.789	2.606	0.502
difficulty	cho	low	26	4.817	2.922	0.573
difficulty	nns	high	27	3.733	2.658	0.511
difficulty	nns	low	26	4.343	2.312	0.453

Solution weight

Table S56. ANOVA Table for solution weight.

dv_type	Cases	Sphericity_Correction	Mean_Squared_Error	df(num)	df(den)	F	p	partial_eta_squared
weight	solution	None	2.97	1	51	56.83	0	0.53
weight	tp	None	1.07	1	51	1.28	0.262	0.02
weight	utility	None	10.17	1	51	1.26	0.266	0.02
weight	solution:utility	None	2.97	1	51	0.12	0.734	0
weight	solution:tp	None	0.38	1	51	2.85	0.097	0.05
weight	tp:utility	None	1.07	1	51	0.12	0.726	0
weight	solution:tp:utility	None	0.38	1	51	0.14	0.714	0

Table S57. Descriptive statistics for solution weight.

dv_type	solution	utility	tp	N	Mean	SD	SE
weight	cho	high	post	27	38.407	1.526	0.294
weight	cho	high	pre	27	38.407	0.844	0.162
weight	cho	low	post	26	38.962	2.323	0.456
weight	cho	low	pre	26	39	2.227	0.437
weight	nns	high	post	27	36.593	2.358	0.454
weight	nns	high	pre	27	36.815	1.733	0.333
weight	nns	low	post	26	36.923	2.058	0.404
weight	nns	low	pre	26	37.308	1.761	0.345

Table S58. Post Hoc Comparisons—SOLUTION.

dv_type	contrast	estimate	SE	df	t.ratio	p.value	cohens_d
weight	cho-nns	1.78	0.237	51	7.539	<.0001	0.934

References

Bigdely-Shamlo, N., Mullen, T., Kothe, C., Su, K.-M., & Robbins, K. A. (2015). The PREP pipeline: standardized preprocessing for large-scale EEG analysis. *Frontiers in Neuroinformatics*, *9*, B153.

Luck, S. J., & Gaspelin, N. (2017). How to get statistically significant effects in any ERP experiment (and why you shouldn't). *Psychophysiology*, *54*(1), 146–157.
